# Comparison of the IDEXX ProCyte One to the ProCyte Dx and ADVIA 120 in Dogs and Cats

**DOI:** 10.1111/vcp.70071

**Published:** 2025-11-04

**Authors:** Richard J. Dulli, Kim Yore, Jeremy Hammond, Dennis B. DeNicola, Mary B. Nabity

**Affiliations:** ^1^ Department of Veterinary Pathobiology School of Veterinary Medicine and Biomedical Science, Texas A&M University College Station Texas USA; ^2^ Department of Comparative, Diagnostic, and Population Medicine College of Veterinary Medicine, University of Florida Gainesville Florida USA; ^3^ IDEXX Laboratories Westbrook Maine USA; ^4^ Laboratory Retrievers, LLC Windham Maine USA

**Keywords:** analyzer, bland–Altman, CBC, hematology, Passing–Bablok

## Abstract

**Background:**

The ProCyte One is a veterinary hematology analyzer. It uses laser flow cytometry with four optical detectors to categorize RBCs, WBCs, and platelets.

**Objective:**

We aimed to compare results from the ProCyte One with the ProCyte Dx and ADVIA 120, as well as the results of manual PCVs and WBC differential counts, to determine their performance for automated CBCs in dogs and cats.

**Materials and Methods:**

ProCyte One inter‐assay and intra‐assay precision were assessed using control material and patient samples. For analyzer comparison, patient samples were run on all three analyzers within 2 h of each other. Passing–Bablok regression and visual inspection of Bland–Altman plots were used to determine analyzer congruence, and Spearman's rank correlation was performed.

**Results:**

Precision was generally excellent, with CV < 4% for most measurands except for reticulocytes, lymphocytes, monocytes, and eosinophils. A total of 140 canine and 96 feline samples were included for the comparison study. Correlations were ≥ 0.93 for all comparisons except mean cell volume (MCV), mean cell hemoglobin concentration (MCHC), reticulocytes, lymphocytes, monocytes, and eosinophils. Statistically significant bias was suggested by Passing–Bablok regression for many measurands, but most were minor except for MCV compared to ADVIA 120, monocytes compared to all methods, and RBC/hematocrit in anemic dogs.

**Conclusions:**

The ProCyte One demonstrates acceptable agreement with ProCyte Dx and ADVIA 120. However, because of the potential for significant differences between methods, operators should exercise caution when comparing patient results between different analyzers.

## 1 Introduction

The automated complete blood count has revolutionized veterinary hematology. Made commercially available in the 1980s, cytometry analyzers provide valuable automated CBC and white blood cell differential results to replace more laborious techniques and supplement manual blood smear review [1, 2]. The two principal technologies used to measure and differentiate blood cells are impedance, on the basis of increased electrical properties as cells pass between two electrodes, and laser flow cytometry, where cells are categorized on the basis of light scatter and, in some analyzers, fluorescent properties. Laser flow cytometric analyzers are increasingly being used in general practice as the technology becomes more widely available in point‐of‐care hematology analyzers.

The IDEXX ProCyte One is a laser flow cytometry analyzer developed by IDEXX Laboratories to be a user‐friendly, in‐clinic option for hematology analysis. New laboratory methods, including hematology analyzers, should be evaluated according to the American Society of Veterinary Clinical Pathology (ASVCP) and International Council for Standardization of Hematology guidelines before their adoption into routine use [3, 4]. As part of this evaluation, the new method should be compared to existing methods to ensure results between the two methods can be directly compared [5]. Comparison is performed by running samples on both methods in parallel to test if results are equivalent, relative to their combined imprecision or to predetermined quality recommendations.

The analyzers chosen for comparison in this study are the ADVIA 120 Hematology System (ADVIA 120) and the ProCyte Dx. The ADVIA 120 was chosen because of its similarity to the ProCyte One with regard to analytical methods and its proven quality and performance in a reference laboratory setting [6, 7, 8]. The ProCyte Dx is similarly proven and is commonly used in both reference laboratory and in‐clinic settings [9]. The ProCyte Dx was chosen as a second comparison method because of its popularity, in addition to important differences in methodology with regard to the evaluation of platelets in dogs and erythrocytes in both dogs and cats.

The goal of this study was to assess the performance of the ProCyte One compared with that of the ProCyte Dx and the ADVIA 120. This was done by measuring precision and comparing observed error to ASVCP guidelines for allowable total error in hematology to determine if results were sufficiently similar that the analyzers may be used interchangeably without affecting clinical interpretation [10]. ProCyte One performance was also compared with manual methods, where applicable, for subjective evaluation of performance.

## 2 Materials and Methods

### 2.1 Sample Collection and Handling

This study was performed using fresh canine and feline EDTA whole blood samples submitted for a CBC to a university teaching hospital clinical pathology laboratory. Residual samples that were submitted from hospital patients were used. Client consent for the use of leftover samples for research is obtained for all patients upon admission. The blood was first processed according to the standard laboratory protocol for a CBC (including analysis with the ADVIA 120) for diagnostic purposes. Next, leftover blood samples were analyzed on the ProCyte Dx and ProCyte One in alternating order on the basis of the calendar day (ProCyte Dx first on even days; ProCyte One first on odd days). Analyzers were operated by either a licensed medical technologist or an unlicensed (but experienced) lab technician. Samples were inverted 10 times immediately before each analyzer run and were run on all three analyzers within 2 h of each other. Samples were excluded if there was gross evidence of clotting, if there was insufficient volume to run the sample on all three analyzers, or if there was a delay of > 2 h between any of the analyzer runs. Additionally, repeat samples from a single patient were excluded to avoid bias. Full blood smear evaluation with a 200‐cell differential was performed on each sample by a single author.

### 2.2 Hematology Analyzers

**FIGURE 1:**
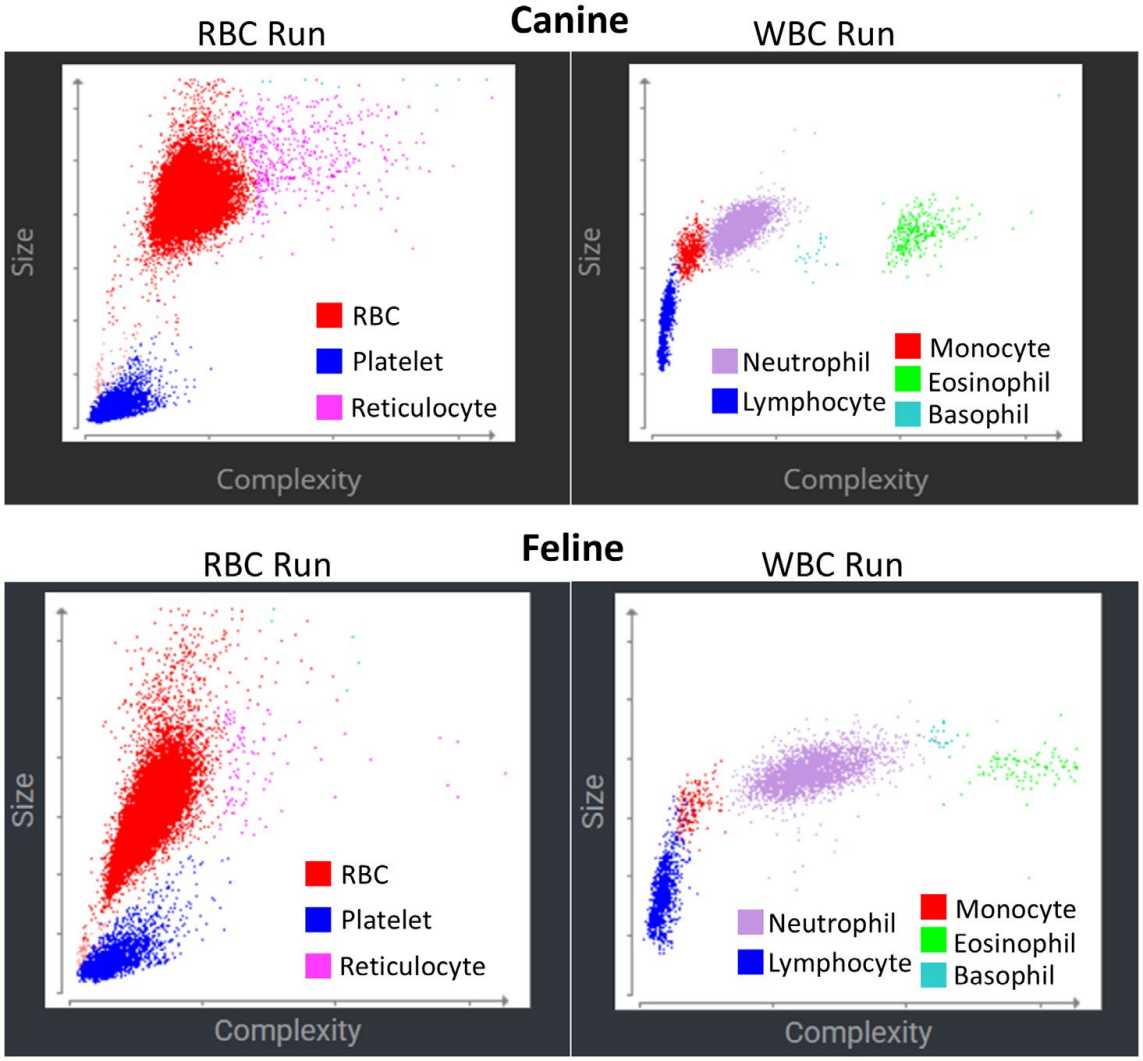
ProCyte One cell scattergrams in a dog and a cat.

The ProCyte One was operated using software version 2.1.1, interfaced through an IDEXX VetLab terminal running version 5.14.038. The instrument is a laser‐based optical flow cytometer with four light scatter detectors: low and high angle forward scatter, side scatter, and extinction [11]. The analyzer categorizes cells on the basis of light scatter properties. The analysis is separated into a red cell analysis and a white cell analysis. RBC and platelets are evaluated with reagents that cause the RBC to sphere. Reagents also contain new methylene blue, which causes staining and aggregation of RNA and increased cytoplasmic complexity in reticulocytes, thereby separating their scatter from mature RBCs. WBC are evaluated in a separate reaction after chemically lysing RBCs, and the 5‐part WBC differential is determined on the basis of scatter properties of the leukocytes. Hemoglobin is measured spectrophotometrically by a cyanide‐free method following lysis of the RBCs. Mean corpuscular volume (MCV) is determined from the RBC data, and hematocrit (HCT), mean corpuscular hemoglobin (MCH), and mean corpuscular hemoglobin concentration (MCHC) are calculated using standard formulas. Normal canine RBC and WBC scattergrams are shown in Figure 1.

Automated quality control for the ProCyte One was performed using SmartQC (IDEXX Laboratories). SmartQC is composed of polymer microbeads suspended in solution that reside in a container on the analyzer, and it is run automatically on a predetermined schedule. The microbeads are analyzed on the basis of light scatter properties similar to cells and are processed as red cell and white cell runs in the same manner as whole blood samples [12]. A SmartQC run within specifications is intended to verify stable operation of optics and microfluidics of the analyzer, although specific quality control run data and criteria used to determine acceptability cannot be directly accessed by the end user. By default, SmartQC is set to run overnight once per week, but for this study, it was configured to run nightly. All SmartQC runs revealed acceptable parameters per instrument logs maintained over the course of the study.

Additionally, the ProCyte One performance was monitored using population‐based analysis QC (PBA‐QC). PBA‐QC is a method of using weighted averages of patient results to look for evidence of analytical drift on the basis that, as long as enough normal patients are included in the analyzed samples, the average result should be close to the middle of the reference interval [13]. The ProCyte One MCV was recalibrated on the basis of PBA‐QC results early in the study period, and all data already collected were adjusted on the basis of the recalibration. This recalibration also affected values calculated on the basis of MCV, including HCT and MCHC.

The ProCyte Dx was operated using software version 00‐35_61, interfaced through the same IDEXX VetLab terminal as the ProCyte One. The ProCyte Dx uses laminar flow impedance as well as flow cytometry for the determination of RBC, HCT, and platelets (PLT) in dogs and cats by chemically sphering RBCs, and a fluorescent dye is applied for reticulocyte detection. For WBC, the system uses optical flow cytometry with chemistry to lyse RBCs and adds a fluorescent dye for the 5‐part WBC differential. Technical specifications have been described in greater detail elsewhere [9]. Quality control material (QCM) was run daily using a fixed blood product (e‐CHECK, IDEXX Laboratories, ME) and was within manufacturer‐specified control limits for the duration of the study. Additionally, as for the ProCyte One, the ProCyte Dx was monitored using PBA‐QC.

The ADVIA 120 was operated using version 6.0. × 121. The ADVIA 120 uses optical flow cytometry through multiple flow channels and myeloperoxidase staining to report a full CBC with a 5‐part differential and reticulocytes. The system functions similarly to other flow cytometer analyzers by separating red cell analyses, with RBC sphering and reticulocyte staining with a nucleic acid dye (Oxazine 750), from the white cell analysis after RBCs have been lysed. Technical specifications have been described in greater detail elsewhere [7, 9]. QCM was run daily using fixed blood products from the manufacturer and was within manufacturer‐specified control limits for the duration of the study. Manufacturer's reported specifications for analytical range and precision for all three analyzers are provided in Table S1.

### 2.3 Precision

Intra‐assay precision was evaluated by running canine and feline samples on the ProCyte One 7 times consecutively for each sample. The mean, standard deviation, and coefficient of variation (CV) for each measurand were calculated. Three canine samples were selected on the basis of specific CBC abnormalities (normal, pancytopenia, and reticulocytosis) in an attempt to capture variation in CV over the analytical range, as recommended by Nabity et al. [10] Only a single feline sample was used because of difficulty acquiring samples with sufficient volume for repeated runs.

Inter‐assay precision was evaluated by running a fixed‐cell control, Para‐12 (Streck, La Vista, NE), with a dedicated algorithm on the ProCyte One once daily over 7 days. Although this is a fixed blood product used for quality control (QC) in some analyzers, it is not designed for accurate targeting of the ProCyte One and cannot be used to evaluate accuracy. For both intra‐assay and inter‐assay precision, a CV less than half of the ASVCP recommendation for total allowable error [10] was considered acceptable, and a CV less than ¼ was considered excellent.

### 2.4 Analyzer Comparison

The following results from the ProCyte One were compared to the ProCyte Dx and ADVIA 120: RBC, HCT, hemoglobin (Hgb), MCV, MCHC, absolute reticulocytes, WBC, absolute counts of neutrophils, lymphocytes, monocytes, and eosinophils, and PLT. Additionally, HCT on the ProCyte One was compared with manual packed cell volume (PCV), and percent neutrophils, lymphocytes, monocytes, and eosinophils were compared to the manual differential. In select cases with major discordances between analyzers, instrument scattergrams and blood smear findings were investigated for possible explanatory factors or evidence of gross laboratory error. Though no results were excluded unless there was irrefutable evidence of major laboratory error, which would invalidate the results (e.g., incorrect patient sample, incorrect species selection, etc.).

ProCyte One, ProCyte Dx, and ADVIA 120 each have logic that evaluates the data from the analyzer and provides flags when the data is presented in a manner where automated confidence is reduced in the reported results. The general result of these flags is one or more parameters that are qualified, meaning that the report indicates those specific parameters should be evaluated further. Flagging data from the ProCyte One and ProCyte Dx was incorporated into figures for visual evaluation, but was not factored into statistical analysis. The ADVIA 120 flag data, though useful, does not clearly demarcate which results are suspect, so ADVIA 120 flags were not considered.

Runs were excluded from comparison of Hgb and MCHC if plasma lipemia or hemolysis exceeded 1+ visually (roughly correlating to lipemia or hemolysis index > 50). Runs were excluded from PLT comparison if greater than three small platelet clumps were seen in the blood smear feathered edge.

### 2.5 Statistical Analysis

All statistical analyses were performed in Microsoft Excel using the Real Statistics add‐in (http://www.real‐statistics.com). Comparison of ProCyte One measurands with the other analyzers was performed by Passing–Bablok regression [14]. The regression line is y = a + bx, and a 95% confidence interval for a (intercept) that did not include 0 and for b (slope) that did not include 1 was considered supportive of constant and proportional bias, respectively. The assumption of linearity was tested by the CUSUM statistic [14]. Parameters that did not meet the assumption of linearity were noted, but were still evaluated by Passing–Bablok regression to maintain consistency in data presentation. Assessment of bias was further evaluated by visual inspection of Bland–Altman plots. Correlation was evaluated by Spearman's rank correlation (*ρ*). Correlations were considered excellent for *ρ* of 0.93–1.00, good for *ρ* of 0.80–0.92, fair for *ρ* of 0.60–0.79, and poor for *ρ* < 0.60 [9]. The difference between the means with 95% limits of agreement (LOA) was calculated for each comparison.

To further evaluate the performance of the ProCyte One compared with each analyzer, total observed error (TEobs) was calculated for RBC, Hgb, MCV, WBC, and PLT and was compared to ASVCP recommendations for total allowable error (TEa) for in‐clinic analyzers [10]. TEobs less than TEa was considered acceptable. TEobs for each analyte was calculated according to the formula:

TEobs=%bias+2CV

where %bias was calculated using the absolute value of the mean bias for each analyte divided by the median value of the data range for the ProCyte Dx or ADVIA 120, respectively, and CV was the inter‐assay CV as described above.

## 3 Results

### 3.1 Precision

Observed precision for the ProCyte One is presented in Table 1. The observed CVs for almost all measurands were acceptable, with most being considered excellent. The only exceptions included reticulocytes in all samples with < 60 000/uL, lymphocytes in all three dogs, and WBC in a single dog (canine 3). In the dog with excessive WBC imprecision, the WBC, lymphocyte, and percent differential results were flagged in 3 out of 7 runs because of a pattern of interference attributed to unlysed RBCs (uRBCs) by the manufacturer. The platelet CV was considerably higher for inter‐assay imprecision compared to intra‐assay, but was still acceptable.

**TABLE 1:** ProCyte One intra‐ and inter‐assay precision.

Measurand	Unit	Acceptable precision (%)	Excellent precision (%)	Canine 1		Canine 2		Canine 3		Feline		Para‐12	
				Mean ± SD	CV (%)	Mean ± SD	CV (%)	Mean ± SD	CV (%)	Mean ± SD	CV (%)	Mean ± SD	CV (%)
RBC	10^12^/L	≤ 5	≤ 2.5	6.50 ± 0.04	0.7	5.99 ± 0.04	0.6	7.73 ± 0.06	0.8	6.99 ± 0.09	1.4	4.46 ± 0.05	1.1
HCT	L/L	≤ 5	≤ 2.5	45.3 ± 0.6	1.3	39.6 ± 0.3	0.7	52.2 ± 0.7	1.3	28.3 ± 0.4	1.5	—	—
Hgb	g/dL	≤ 5	≤ 2.5	14.5 ± 0.2	1.4	13.3 ± 0.2	1.5	17.8 ± 0.3	1.7	9.1 ± 0.2	2.4	9.57 ± 0.19	2.0
MCV	fL	≤ 3.5	≤ 1.75	69.8 ± 0.7	0.9	66.2 ± 0.5	0.8	67.5 ± 0.4	0.6	40.6 ± 0.3	0.8	59.4 ± 0.8	1.3
MCHC	g/dL	≤ 5	≤ 2.5	32.1 ± 0.4	1.3	33.7 ± 0.5	1.3	34.0 ± 0.4	1.1	32.0 ± 0.9	2.7	—	—
Reticulocytes	10^9^/L	≤ 10	≤ 5	32.6 ± 3.4	10.5	22.9 ± 5.2	23.8	96.2 ± 5.7	6.0	6.3 ± 1.1	16.7	—	—
WBC	10^9^/L	≤ 10	≤ 5	8.9 ± 0.1	1.5	3.7 ± 0.1	2.0	5.15 ± 0.6^a^	11.6^a^	19.9 ± 0.3	1.7	4.72 ± 0.12^b^	2.5^b^
Neutrophils	10^9^/L	≤ 7.5	≤ 3.75	8.0 ± 0.1	1.3	2.8 ± 0.06	2.3	1.6 ± 0.06	3.7	16.7 ± 0.2	1.4	—	—
Lymphocytes	10^9^/L	≤ 7.5	≤ 3.75	0.40 ± 0.04	9.1	0.42 ± 0.05	10.8	2.69 ± 0.62^a^	22.9^a^	1.45 ± 0.06	4.0	—	—
Monocytes	10^9^/L	≤ 30	≤ 15	0.46 ± 0.03	6.1	0.48 ± 0.02	3.9	0.20 ± 0.03	12.9	1.39 ± 0.06	4.4	—	—
Eosinophils	10^9^/L	≤ 25	≤ 12.5	0.10 ± 0.01	12.9	0.05 ± 0.01	26.2	0.54 ± 0.10	18.1	0.34 ± 0.03	8.8	—	—
Platelets	10^9^/L	≤ 12.5	≤ 6.25	307 ± 7	2.5	95 ± 3	3.5	349 ± 7	2.1	394 ± 13	3.4	228 ± 15	6.9

*Note:* Canine and feline samples were run 7 times consecutively to assess intra‐assay precision. Para‐12, a commercially available fixed blood product, was run daily over 7 days to measure inter‐assay precision. Precision was judged by comparing CV to the ASVCP recommendation for allowable total error in‐clinic analyzers (TEa) [10]. CV ≤ ½ TEa was considered acceptable. CV ≤ ¼ TEa was considered excellent.

Abbreviations: CV, coefficient of variation; HCT, hematocrit; Hgb, hemoglobin; MCHC, mean corpuscular hemoglobin concentration; MCV, mean cell volume; RBC, red blood cells; SD, standard deviation; WBC, white blood cells.

^a^ There was repeatable excess interference from unlysed RBCs appreciated in the WBC scattergram. The interference affected the WBC count, lymphocytes, and WBC differential. These values were flagged by the instrument in 3 out of 7 runs.

^b^ White blood cells excluding lymphocytes because of interference from poor RBC lysis in fixed blood.

### 3.2 Analyzer Comparisons

#### 3.2.1 Canine

A CBC was performed on all three analyzers in 140 dogs. Three samples were excluded from the WBC comparisons, as the ProCyte One did not report WBC parameters because of excessive interference from unlysed RBCs. Ten samples were excluded from the comparison of Hgb and MCHC because of interference from hemolysis or lipemia. Twenty‐three samples were excluded from the platelet comparison because of clumping on the blood film. A manual PCV was not available in one sample. Full descriptive statistics of included canine samples are in Table S2.

Correlation was excellent for RBC, HCT, Hgb, WBC, neutrophils, and platelets compared to both analyzers and for eosinophils compared to ProCyte Dx (Table 2). Correlation was good for MCV, reticulocytes, and monocytes compared to both analyzers and for eosinophils compared to ADVIA 120. Correlation with both analyzers was fair for lymphocytes and poor for MCHC.

**TABLE 2:** Comparison of the ProCyte One automated CBC results with the ProCyte DX and ADVIA 120 in dogs.

Measurand	Unit	*n*	ProCyte Dx				ADVIA 120			
			*ρ*	Slope (95% CI)	Intercept (95% CI)	Bias (95% LOA)	*ρ*	Slope (95% CI)	Intercept (95% CI)	Bias (95% LOA)
RBC	10^12^/L	140	0.99	**1.04** ^a^ **(1.02–1.07)**	**−0.2** ^a^ **(−0.3, −0.03)**	0.1 (−0.2, 0.4)	0.99	**1.11** ^a^ **(1.08–1.14)**	**−0.5** ^a^ **(−0.7, −0.4)**	0.1 (−0.3, 0.6)
HCT	L/L	140	0.98	0.99^a^ (0.96–1.03)	1.3^a^ (−0.2, 2.6)	0.8 (−2.5, 4.1)	0.99	**1.04 (1.01–1.07)**	**−4.4 (−5.6, −2.9)**	−2.6 (−5.8, 0.5)
Hgb	g/dL	130	0.99	1.00 (0.97–1.01)	0.2 (0.0, 0.6)	0.2 (−0.5, 0.9)	0.99	1.03 (1.00–1.06)	−0.3 (−0.8, 0.1)	0.0 (−0.8, 0.9)
MCV	fL	140	0.83	0.95 (0.87–1.04)	3.4 (−2.3, 8.8)	0.3 (−3.2, 3.7)	0.87	1.03 (0.95–1.12)	**−8.0 (−13.9, −2.0)**	−6.0 (−9.7, −2.3)
MCHC	g/dL	130	0.11	1.05 (0.75–1.58)	−2.2 (−20.5, 8.6)	−0.1 (−3.9, 3.7)	0.01	**1.58** ^a^ **(1.05, 2.32)**	−17.0^a^ (−41.1, 0.3)	2.4 (−1.6, 6.5)
Retic	10^9^/L	140	0.90	**0.84** ^a^ **(0.80–0.89)**	**−2.6** ^a^ **(−4.4, −0.3)**	−11 (−53, 31)	0.89	**0.88** ^a^ **(0.82, 0.95)**	**−9.2** ^a^ **(−12.5, −6.4)**	−17 (−78, 45)
WBC	10^9^/L	137	0.99	1.00 (0.98–1.02)	−0.1 (−0.3, 0.1)	0.0 (−5.9, 5.8)	0.99	1.03 (1.00–1.05)	0.0 (−0.3, 0.2)	0.3 (−6.1, 6.8)
Neutrophils	10^9^/L	137	0.98	1.00 (0.99–1.02)	**−0.2 (−0.4, −0.1)**	−0.1 (−2.4, 2.1)	0.96	1.02 (1.00–1.03)	0.0 (−0.1, 0.1)	0.1 (−2.1, 2.2)
Lymph	10^9^/L	137	0.78	1.09 (0.98–1.24)	−0.1 (−0.4, 0.0)	0.4 (−5.4, 6.3)	0.72	1.04 (0.93–1.09)	−0.2 (−0.4, 0.0)	−0.1 (−5.6, 5.5)
Monocytes	10^9^/L	137	0.91	**1.32** ^a^ **(1.23–1.47)**	0.0^a^ (−0.1, 0.1)	−0.2 (−7.8, 7.3)	0.91	**2.05 (1.91–2.21)**	**−0.2 (−0.3, −0.1)**	0.5 (−2.1, 3.1)
Eosinophils	10^9^/L	137	0.95	**0.88** ^a^ **(0.84–0.91)**	0.0^a^ (0.0, 0.0)	−0.07 (−0.4, 0.2)	0.89	1.02 (0.97–1.07)	0.0 (0.0, 0.0)	−0.03 (−0.2, 0.2)
Platelets	10^9^/L	117	0.99	**0.84 (0.81–0.87)**	**14 (4, 22)**	−38 (−118, 42)	0.97	**0.93** ^a^ **(0.89–0.98)**	11^a^ (−1, 20)	−16 (−110, 79)

*Note:* Bolded results for Slope indicate the 95% CI does not include 1, suggesting proportional bias is present. Bolded results for Intercept indicate the 95% CI does not include 0, suggesting constant bias is present.

Abbreviations: CI, confidence interval; HCT, hematocrit; Hgb, hemoglobin; LoA, limits of agreement; MCHC, mean corpuscular hemoglobin concentration; MCV, mean cell volume; *n*, number of samples; RBC, red blood cells; WBC, white blood cells; *ρ*, Spearman's rho.

^a^ Indicates residuals are unevenly distributed on the basis of the CUSUM statistic, suggesting data is not linear and Passing–Bablok regression may be inappropriate. Results should be interpreted with caution.

**FIGURE 2:**
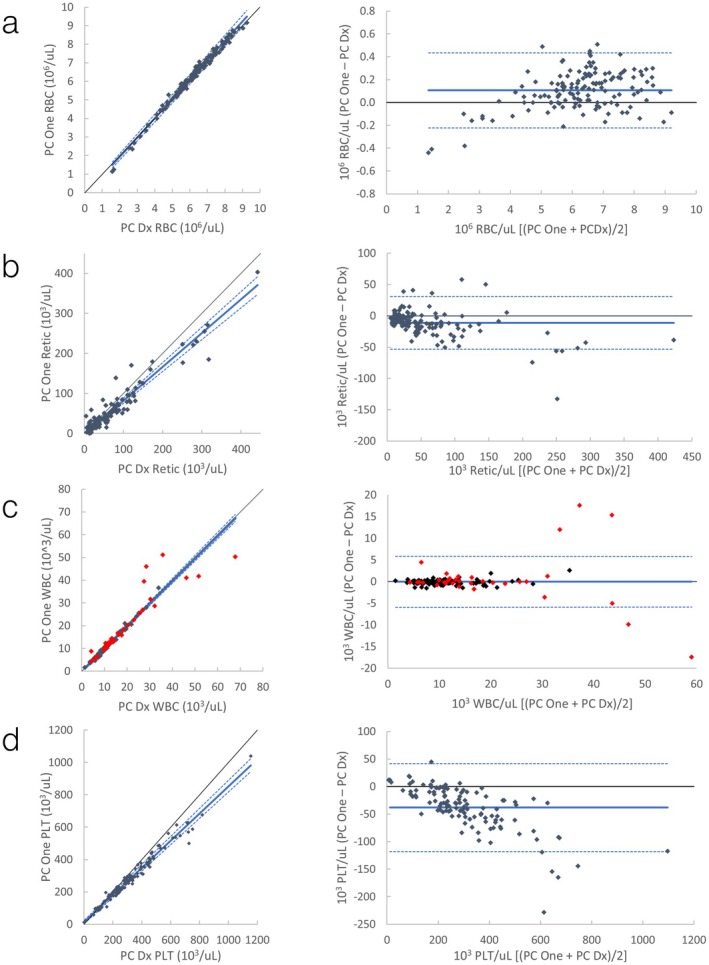
Canine automated CBC comparison between ProCyte One (PC One) and ProCyte Dx (PC Dx). *Left graphs*: Scatter plots with Passing–Bablok regression line (solid blue line) and confidence interval (dashed blue lines). Black is a line of identity. *Right graphs*: Bland–Altman plots with mean (solid blue line) +/− 1.96 SD (dashed blue lines). (a) Comparison of automated RBC; (b) Comparison of automated absolute reticulocytes; (c) Comparison of automated WBC. Red data points are runs where WBC was flagged by the ProCyte One, the ProCyte Dx, or both; (d) Comparison of automated platelets.

**FIGURE 3:**
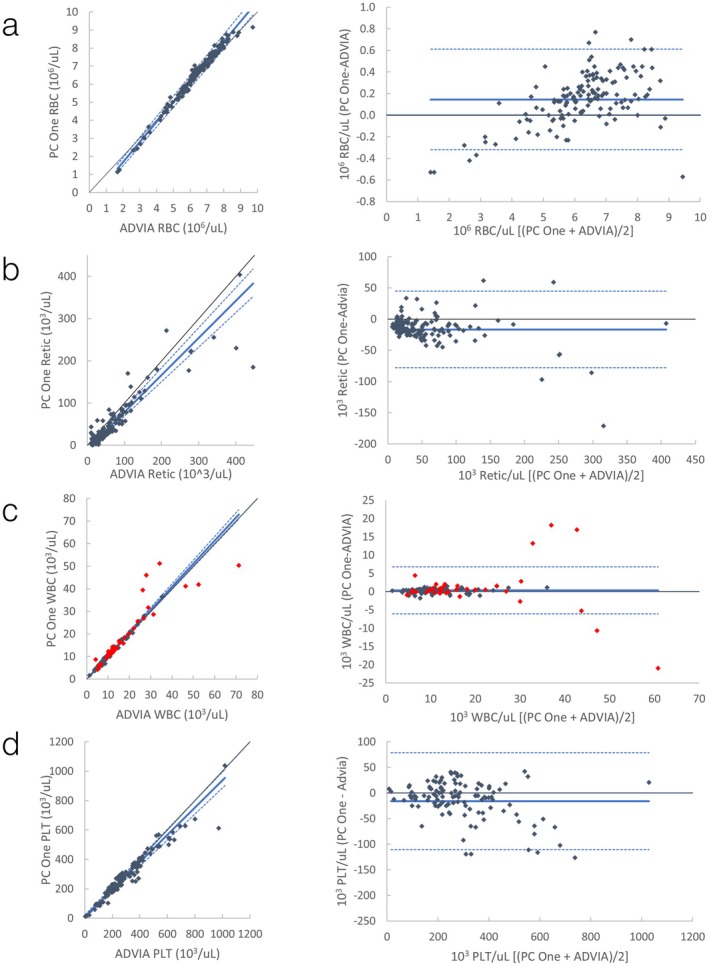
Canine automated CBC comparison between ProCyte One (PC One) and ADVIA 120 (ADVIA). *Left graphs*: Scatter plots with Passing–Bablok regression line (solid blue line) and confidence interval (dashed blue lines). Black is line of identity. *Right graphs*: Bland–Altman plots with mean (solid blue line) +/− 1.96 SD (dashed blue lines). (a) Comparison of automated RBC; (b) Comparison of automated absolute reticulocytes; (c) Comparison of automated WBC. Red data points are runs where WBC was flagged by the ProCyte One, the ProCyte Dx, or both; (d) Comparison of automated platelets.

Passing–Bablok regression statistics for comparison to ProCyte Dx and ADVIA 120 are shown in Table 2, and comparison plots are presented in Figures 2 and 3; Figures S1–S4. Comparisons that failed the assumption of linearity included HCT, monocytes, and eosinophils compared to ProCyte Dx, MCHC and PLT compared to ADVIA 120, and RBC and reticulocytes compared to both. Most observed biases were subjectively mild, with the exceptions of a moderate (ProCyte Dx) to large (ADVIA 120) positive proportional bias for monocytes (Figures S3c and S4c) and a large negative constant bias for MCV compared with ADVIA 120 (Figure S2c). Additionally, on the basis of visual inspection, agreement for RBC was close over most of the range, but the ProCyte One appeared to consistently and proportionally underestimate this value compared with both analyzers in the limited number (13/139) of dogs with PCV < 30% (Figures 2a and 3a).

On the ProCyte One, WBC was flagged for unreliability in 34/140 samples. In 25 of the 34 flagged runs, this flag corresponded to the classification and display of uRBCs in the instrument WBC run, as uRBCs overlap with the position of lymphocytes, suggesting a potential false increase in both WBC and lymphocyte counts. The cause for the instrument flag was not always clear in the remaining 9 samples, but interference from marked platelet clumping or lipemia may have played a role in some. To evaluate the significance of the presumed interference from uRBCs, ProCyte One WBC was compared to the average of the two other analyzers for these 25 samples. The ProCyte One WBC had a positive mean bias of 4%, with > 15% positive bias in only 3/25 of these samples. Additionally, an association was seen between interference from uRBCs on the ProCyte One and the “bands suspected” flag on the ProCyte Dx (Fisher's exact *p* < 0.00001). Though it did not affect the absolute WBC count, the absolute neutrophil, lymphocyte, and monocyte counts and the leukocyte differential were flagged on the ProCyte Dx in 16/137 samples in a pattern consistent with previously reported interference from increased fluorescence in toxic or immature neutrophils [15].

Although instrument flags and examination of the instrument scattergrams provided clear evidence of interference in some patients, these samples were not excluded from statistical analysis, as both Passing–Bablok regression and Spearman's correlation are robust to outliers [14]. Thus, excluding samples with evidence of interference had minimal effects on the regression line or Spearman's rho (Table S4). Results flagged by the ProCyte One (and/or ProCyte Dx, where applicable) were indicated in red in the provided figures for ease of visual inspection.

#### 3.2.2 Feline

Ninety‐six feline samples were included. One sample was excluded from comparison to ProCyte Dx because of an invalid run. Two samples did not have a manual PCV available. Three samples were excluded from the Hgb and MCHC comparison because of interference from hemolysis or lipemia. A blood smear was not available in seven samples, which were excluded from comparison to the manual differential and platelet comparison, as platelet clumping could not be evaluated. An additional 32 samples were excluded from platelet comparison because of clumping. Only 5 out of 96 cats had > 60 000 reticulocytes, so reticulocyte comparison was not done. However, there was good agreement for the one strongly regenerative feline sample, with the reported reticulocytes on the three analyzers ranging from 150 700 to 191 300/μL. Full descriptive statistics of included feline samples are presented in Table S3.

Table 3 shows correlation, bias, and regression statistics compared with the ProCyte Dx and ADVIA 120. There was excellent correlation for RBC, HCT, Hgb, WBC, neutrophils, and platelets with both analyzers and for eosinophils with the ProCyte Dx. Correlation was good to fair for all other analytes, with the exception of MCHC, which was poor. Overall bias was < 1% for HCT compared with both analyzers, but there was relatively wide variance in the biases of individual samples as evidenced by the wide 95% LOA, spanning from −7.5% to 7.1% bias in the case of ProCyte One compared with the ProCyte Dx (Table 3). The same was also true for MCV compared to ProCyte Dx, where the overall bias was only −1.6 fL, but the 95% LOA was −9.4 fL to 6.2 fL, and individual runs had up to a 14.3 fL difference between the two analyzers.

**TABLE 3:** Comparison of the ProCyte One automated CBC results with the ProCyte DX and ADVIA 120 in cats.

Measurand		ProCyte Dx					ADVIA 120				
	Unit	*n*	*ρ*	Slope (95% CI)	Intercept (95% CI)	Bias (95% LOA)	*n*	*ρ*	Slope (95% CI)	Intercept (95% CI)	Bias (95% LOA)
RBC	10^12^/L	95	0.98	0.99 (0.96–1.01)	**0.4 (0.2, 0.6)**	0.2 (−0.6, 1.1)	96	0.99	**1.06 (1.04–1.08)**	0.0 (−0.2, 0.1)	0.4 (−0.3, 1.1)
HCT	L/L	95	0.93	0.94 (0.86–1.01)	2.8 (−0.1, 5.6)	−0.2 (−7.5, 7.1)	96	0.97	1.04 (0.98–1.09)	−1.4 (−3.2, 0.6)	−0.6 (−5.6, 4.4)
Hgb	g/dL	92	0.99	1.01 (0.99–1.05)	0.1 (−0.4, 0.3)	0.2 (−0.8, 1.2)	93	0.99	**1.07 (1.04–1.11)**	**−0.7 (−1.1, −0.3)**	0.1 (−0.8, 1.1)
MCV	fL	95	0.74	1.03 (0.87–1.23)	−2.3 (−11.5, 4.8)	−1.6 (−9.4, 6.2)	96	0.91	**1.30 (1.20–1.41)**	**−16.5 (−21.4, −11.7)**	−2.8 (−7.0, 1.4)
MCHC	g/dL	92	−0.33	1.67^a^ (0.87–3.24)	−21.4^a^ (−72.0, 4.4)	0.6 (−5.6, 6.9)	93	−0.2	^b^	^b^	0.8 (−4.2, 5.9)
WBC	10^9^/L	95	0.96	1.00 (0.96–1.04)	**−0.4 (−0.8, −0.1)**	−0.6 (−4.2, 2.9)	96	0.94	1.02^a^ (0.98–1.07)	**−0.5** ^a^ **(−0.9, −0.1)**	−0.5 (−4.3, 3.4)
Neutrophils	10^9^/L	95	0.95	1.00 (0.97–1.02)	**−0.2 (−0.3, −0.1)**	0.3 (−6.3, 6.8)	96	0.95	1.00^a^ (0.97–1.03)	**−0.3** ^a^ **(−0.5, −0.1)**	−0.4 (−2.8, 2.1)
Lymph	10^9^/L	95	0.74	**0.80 (0.68–0.93)**	0.0 (−0.2, 0.3)	−0.9 (−6.3, 4.5)	96	0.82	0.91 (0.80–1.03)	−0.2 (−0.5, 0.0)	−0.5 (−3.0, 2.1)
Monocytes	10^9^/L	95	0.83	**1.40 (1.25–1.56)**	0.0 (0.0, 0.1)	0.1 (−1.0, 1.3)	96	0.79	**2.59 (2.24–2.98)**	−0.1 (−0.1, 0.0)	0.3 (−0.3, 1.0)
Eosinophils	10^9^/L	95	0.93	0.97 (0.93–1.01)	0.0 (0.0, 0.0)	0.0 (−0.2, 0.2)	96	0.82	1.08 (0.95–1.22)	0.0 (0.0, 0.1)	0.04 (−0.3, 0.3)
Platelets	10^9^/L	56	0.97	**0.91 (0.85–0.96)**	3.9 (−8.9, 16.3)	−25 (−81, 31)	57	0.97	1.07 (1.00–1.14)	−9.0 (−27.9, 1.0)	2 (−56, 59)

*Note:* Bolded results for Slope indicate the 95% CI does not include 1, suggesting proportional bias is present. Bolded results for Intercept indicate the 95% CI does not include 0, suggesting constant bias is present.

Abbreviations: CI, confidence interval; HCT, hematocrit; Hgb, hemoglobin; LoA, limits of agreement; MCHC, mean corpuscular hemoglobin concentration; MCV, mean cell volume; *n*, number of samples; RBC, red blood cells; WBC, white blood cells; *ρ*, Spearman's rho.

^a^ Indicates residuals are unevenly distributed on the basis of the CUSUM statistic, suggesting data are not linear and Passing–Bablok regression may be inappropriate. Results should be interpreted with caution.

^b^ A regression line could not be calculated because of poor correlation and a short data range.

**FIGURE 4:**
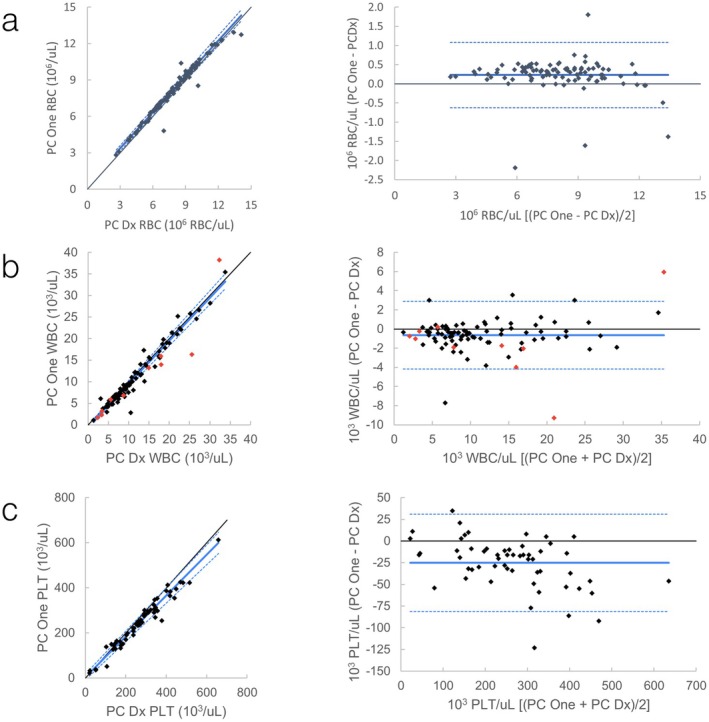
Feline automated CBC comparison between ProCyte One (PC One) and ProCyte Dx (PC Dx). *Left graphs*: Scatter plots with Passing–Bablok regression line (solid blue line) and confidence interval (dashed blue lines). Black is line of identity. *Right graphs*: Bland–Altman plots with mean (solid blue line) +/− 1.96 SD (dashed blue lines). (a) Comparison of automated RBC; (b) Comparison of automated WBC. Red data points are runs where WBC was flagged by the ProCyte One, the ProCyte Dx, or both; (c) Comparison of automated platelets.

**FIGURE 5:**
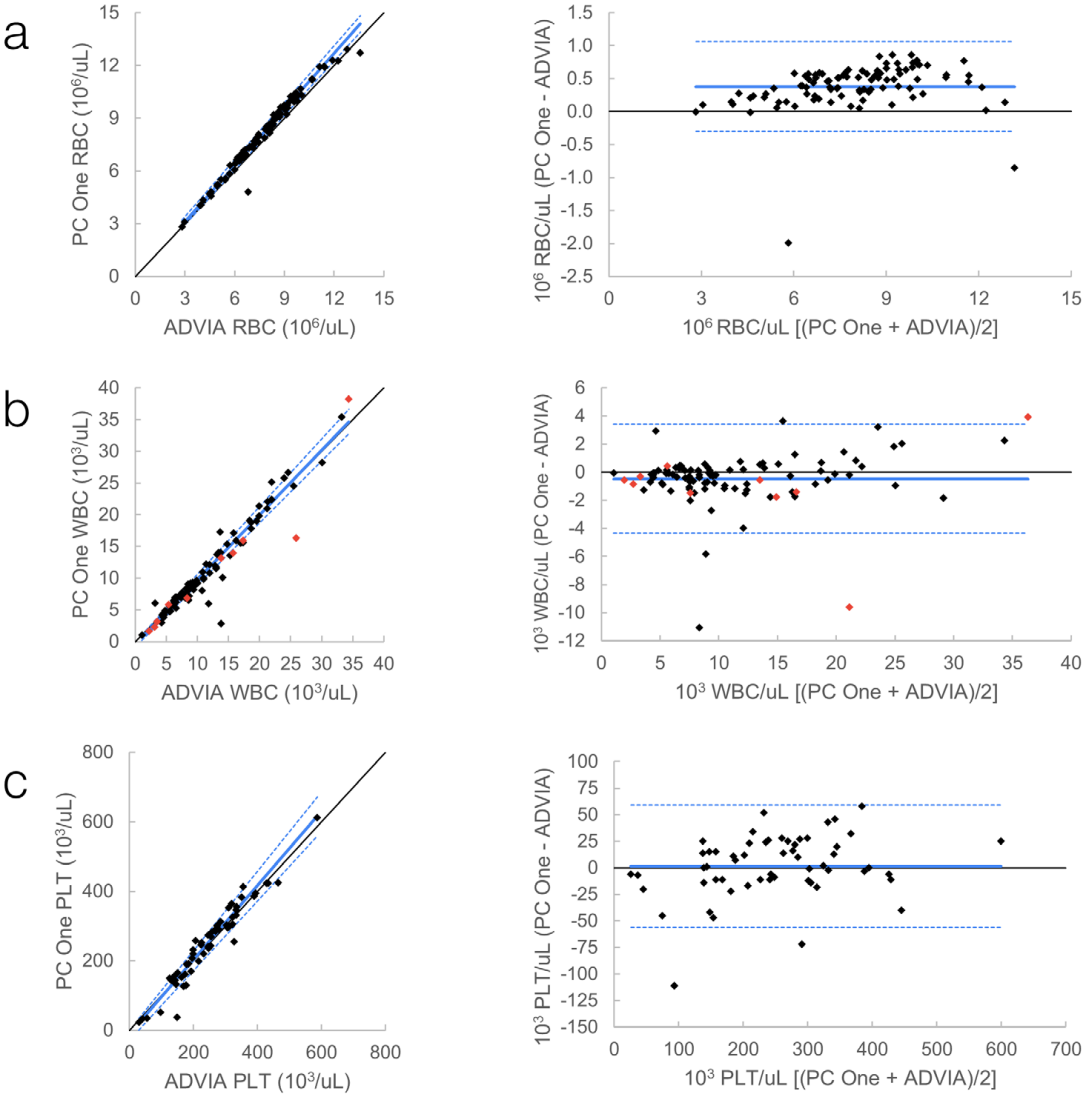
Feline automated CBC comparison between ProCyte One (PC One) and ADVIA 120 (ADVIA). *Left graphs*: Scatter plots with Passing–Bablok regression line (solid blue line) and confidence interval (dashed blue lines). Black is line of identity. *Right graphs*: Bland–Altman plots with mean (solid blue line) +/− 1.96 SD (dashed blue lines). (a) Comparison of automated RBC; (b) Comparison of automated WBC. Red data points are runs where WBC was flagged by the ProCyte One, the ProCyte Dx, or both; (c) Comparison of automated platelets.

Results of Passing–Bablok regression and Bland–Altman plots are shown in Table 3 and Figures 4 and 5; Figures S5–S8. Comparisons that failed the assumption of linearity included WBC and neutrophils compared with the ADVIA 120 and MCHC compared with both the ProCyte Dx and ADVIA 120. The most notable observed bias was in the comparison of feline monocytes to ADVIA 120, with a regression slope of > 2, suggesting large proportional bias (Figure S8c).

For the ProCyte One, WBC was flagged in only 3/96 feline samples, none of which had evidence of uRBC interference. The ProCyte Dx flagged WBC in 10/95 samples. As in the dog samples, the cause of WBC flags in both instruments was not always clear, but platelet clumping likely contributed in some cases. Additionally, spurious decreases in neutrophils and increases in lymphocytes and monocytes because of inflammation were seen on the ProCyte Dx in 5/95 samples.

### 3.3 Comparison to Manual Methods

**FIGURE 6:**
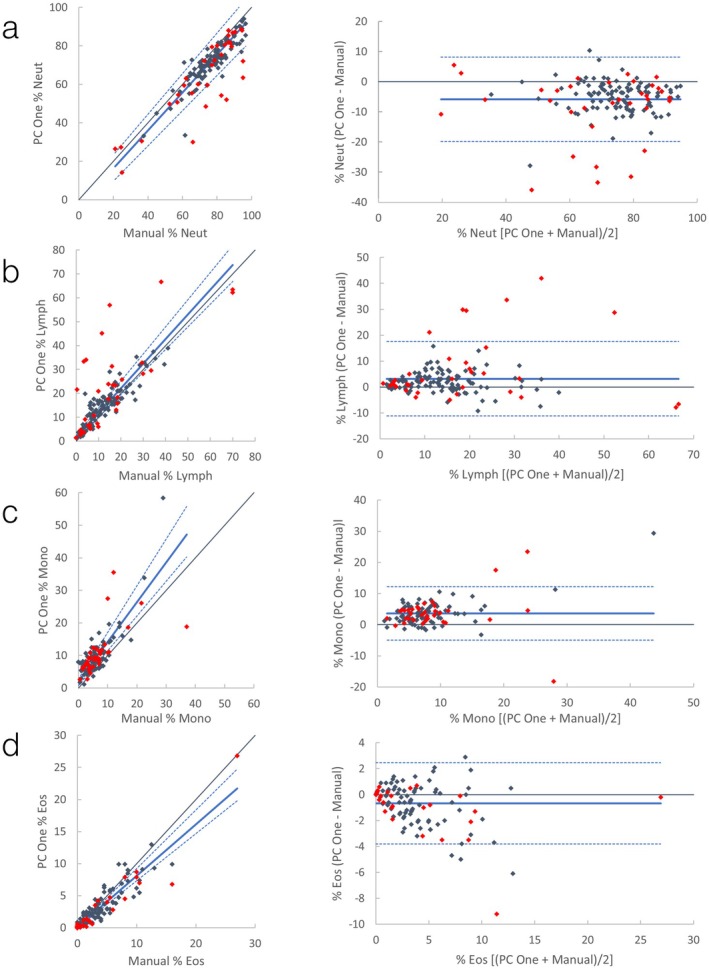
Canine WBC differential comparisons between ProCyte One and a 200‐cell manual blood smear count. *Left graphs*: Scatter plots with Passing–Bablok (solid blue line) and confidence interval (dashed blue lines). Black is line of identity. *Right graphs*: Bland–Altman plots with mean (solid blue line) +/− 1.96 SD (dashed blue lines). Red data points are runs where the WBC differential was flagged by the ProCyte One. Flagged runs are included in regression lines and descriptive statistics. (a) Comparison of % neutrophils; (b) Comparison of % lymphocytes; (c) Comparison of % monocytes; (d) Comparison of % eosinophils.

**FIGURE 7:**
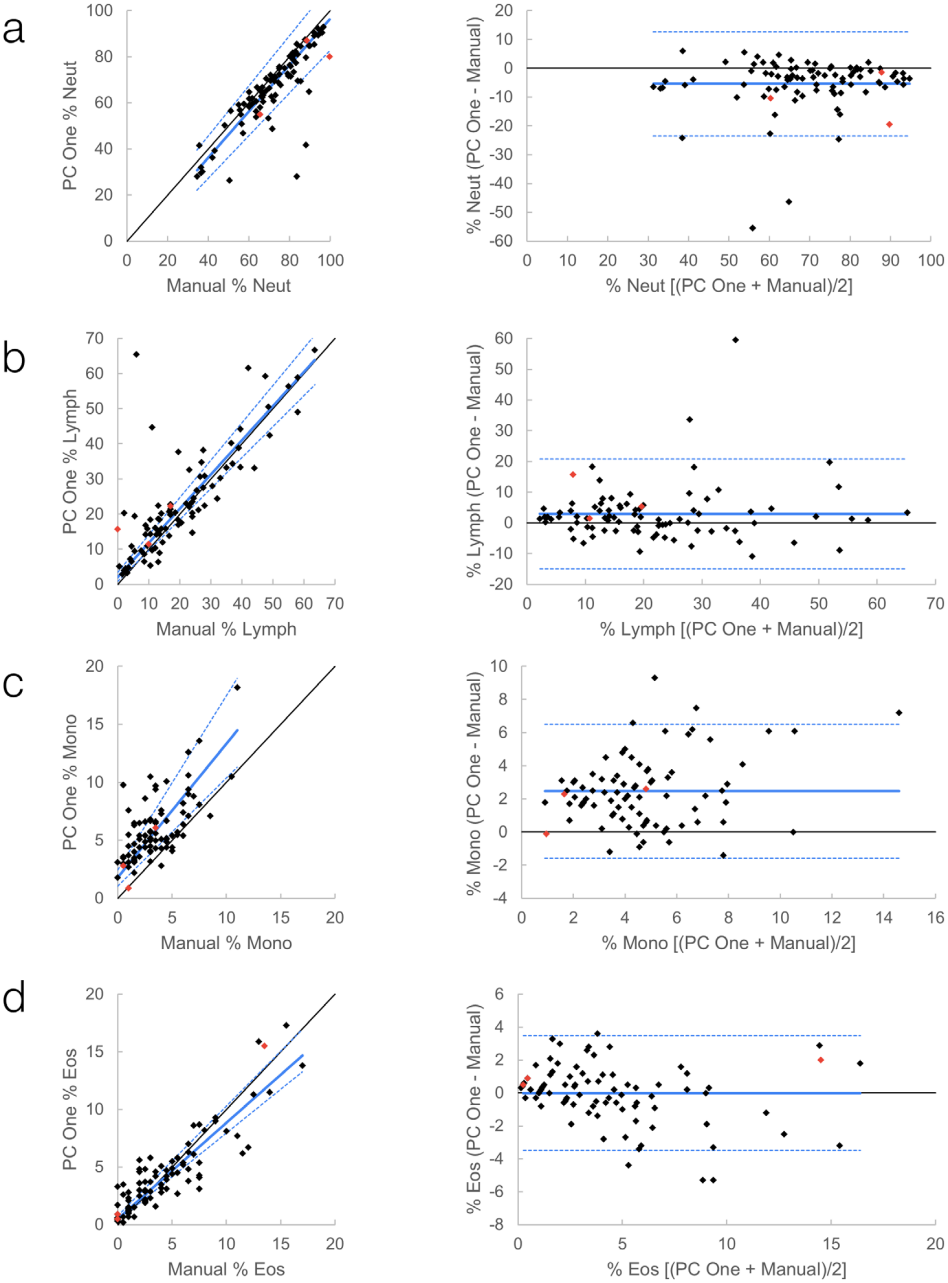
Feline WBC differential comparisons between ProCyte One and a 200‐cell manual blood smear count. *Left graphs*: Scatter plots with Passing–Bablok (solid blue line) and confidence interval (dashed blue lines). Black is line of identity. *Right graphs*: Bland–Altman plots with mean (solid blue line) +/− 1.96 SD (dashed blue lines). Red data points are runs where the WBC differential was flagged by the ProCyte One. Flagged runs are included in regression lines and descriptive statistics. (a) Comparison of % neutrophils; (b) Comparison of % lymphocytes; (c) Comparison of % monocytes; (d) Comparison of % eosinophils.

Statistics for the comparison of the ProCyte One to manual methods are in Table 4, and comparison plots are shown in Figures 6 and 7; Figures S9 and S10. Correlation of the ProCyte One HCT to the manual PCV was excellent overall in both species. Correlation compared with the manual WBC differential was good for neutrophils and lymphocytes, fair for monocytes, and excellent (canine) to good (feline) for eosinophils. Bias was suggested by Passing–Bablok regression for lymphocytes, monocytes, and eosinophils for both species, and for the HCT in the dog compared with manual methods, but these differences were subjectively mild on the basis of evaluation of comparison plots (Figures 6 and 7). Although the tendency toward negative bias of the RBCs seen in anemic dogs in the analyzer comparison, it was also appreciated compared with PCV, with some relatively large and potentially clinically important biases in individual runs (Figure S9a). The ADVIA HCT was compared with the manual PCV in dogs to help investigate the large positive bias in MCVs as compared with the ProCyte One. ADVIA HCT had a mean positive bias of 2.7% compared with the manual PCV (Figure S9c).

**TABLE 4:** Comparison of the ProCyte One with the manual PCV and 200‐cell WBC differential.

Measurand	Canine					Feline				
	*n*	*ρ*	Slope (95% CI)	Intercept (95% CI)	Bias (95% LoA)	*n*	*ρ*	Slope (95% CI)	Intercept (95% CI)	Bias (95% LoA)
PCV	139	0.98	**1.10 (1.06–1.13)**	**−3.8 (−5.3, −2.1)**	0.1 (−3.9, 4.0)	94	0.96	1.03 (0.97–1.09)	0.2 (−1.9, 2.0)	1.0 (−4.1, 6.0)
% Neutrophil	137	0.85	0.98 (0.92–1.06)	−3.3 (−8.7, 2.2)	−5.9 (−19.9, 8.1)	88	0.85	1.01 (0.93–1.09)	−4.1 (−9.8, 2.0)	−5.4 (−23.4, 12.6)
% Lymphocyte	137	0.81	1.03^a^ (0.95–1.14)	**1.4** ^a^ **(0.5, 2.5)**	3.2 (−11.2, 17.6)	88	0.83	0.97 (0.88–1.07)	**2.1 (0.9, 3.4)**	3.0 (−14.9, 20.8)
% Monocyte	137	0.71	**1.22 (1.07–1.43)**	**1.9 (0.8–2.8)**	3.6 (−5.0, 12.2)	88	0.61	1.15 (0.93–1.50)	**1.8 (1.1, 2.5)**	2.5 (−1.6, 6.5)
% Eosinophil	137	0.93	**0.80 (0.74–0.91)**	0.1 (0.0–0.1)	−0.7 (−3.8, 2.5)	88	0.86	**0.83 (0.76–0.95)**	**0.5 (0.3, 0.9)**	0.0 (−3.5, 3.5)

*Note:* Bolded results for Slope indicate the 95% CI does not include 1, suggesting proportional bias is present. Bolded results for Intercept indicate the 95% CI does not include 0, suggesting constant bias is present.

Abbreviations: CI, confidence interval; LoA, limits of agreement; *n*, number of samples; PCV, packed cell volume; *ρ*, Spearman's rho.

^a^ Indicates residuals are unevenly distributed, suggesting data is not linear and Passing–Bablok regression may be inappropriate. Results should be interpreted with caution.

### 3.4 Performance Relative to Quality Goals

Performance relative to TEa compared to ProCyte Dx and ADVIA 120 is shown in Table 5. ProCyte One had TEobs that exceeded TEa for MCV compared with the ADVIA 120 in both cats and dogs, and for platelets compared with the ProCyte Dx in dogs.

**TABLE 5:** ProCyte One performance compared with the ProCyte Dx and ADVIA 120 relative to ASVCP recommended total allowable error (TEa).

Measurand	TEa^a^ (%)	CV (%)	ProCyte Dx				ADVIA 120			
			Canine		Feline		Canine		Feline	
			Bias (%)	TEobs (%)	Bias (%)	TEobs (%)	Bias (%)	TEobs (%)	Bias (%)	TEobs (%)
RBC	10	1.1	1.7	3.9	2.9	5.1	2.4	4.6	4.8	7.0
Hgb	10	2.0	1.4	5.4	1.4	5.4	0.3	4.3	0.9	4.9
MCV	7	1.3	0.4	3.0	3.4	6.0	8.4	**11.0**	6.0	**8.6**
WBC	20	2.5	0.1	5.1	6.8	11.8	2.5	7.5	5.7	10.7
Platelets	25	6.9	12.5	**26.3**	10.3	24.1	5.2	19.0	0.7	14.5

*Note:* Bolded results indicate TEobs greater than TEa.

Abbreviations: CV, Inter‐assay coefficient of variation; HCT, hematocrit; Hgb, hemoglobin; MCV, mean cell volume; RBC, red blood cells; TEa, total allowable error; TEobs, total observed error; WBC, white blood cells.

^a^ On the basis of ASVCP guidelines for allowable total error in hematology [10].

## 4 Discussion

This study evaluated the performance of the ProCyte One compared with the ProCyte Dx and Advia 120 for automated CBCs in dogs and cats. For most analytes, the ProCyte One demonstrated good analytical performance, with high precision and results comparable to the other analyzers. The most notable exceptions were MCV compared with the ADVIA 120 and platelets compared to the ProCyte Dx. Additionally, major bias was noted for monocytes compared with both analyzers, and minor biases were observed for several other measurands.

The ProCyte One had excellent precision, with the observed CV for the majority of parameters around 2% or less (Table 1). The higher CV for reticulocytes is consistent with what has been reported for other analyzers [9, 16], especially when reticulocytes are less than 60 000/μL, where a high degree of imprecision is expected and is not considered clinically relevant [10]. In contrast, unacceptably high imprecision for lymphocytes was observed across all three dogs. Two of these dogs (Canine 1 and 2) were lymphopenic, with lymphocytes < 500/μL in each (ProCyte One RI 1.05–5.10 K/μL). These low absolute values likely contributed to the high imprecision, as even a small amount of absolute error can result in a large percent error. However, further investigation is warranted, ideally in dogs with lymphocytes near the low end of the reference interval, as this value routinely functions as a determining factor in the identification of a stress leukogram. The excess imprecision in the lymphocytes (and WBC) of the third dog (Canine 3) is explained by “uRBCs”. This interference was flagged in 3 of the 7 repeated runs, but could be appreciated visually in the dot plot, even in the 4 runs where it was not flagged. It is unclear why there is incomplete RBC lysis in some samples, but the repeatability of this finding in a single sample suggests it is a sample‐related property. This corroborates impressions from IDEXX internal testing, where repeatable incomplete RBC lysis has been anecdotally appreciated in some dogs (D.B. DeNicola, personal communication).

As noted, this interference was also seen in the comparison study, affecting 18% of canine samples. Though common, this interference was generally mild. Only 3 samples had greater than 15% error for WBC, which is the ASVCP recommendation for total allowable error [10]. Thus, clinically significant interference from uRBCs appears uncommon, particularly if the automated differential is similar to the manual differential. However, practitioners should be aware of the possibility of spuriously increased WBC and lymphocytes in canine patients.

Although the cause of the excess unlysed RBCs in some dogs is uncertain, the association between interference from uRBCs and the “Band suspected” flag on the ProCyte Dx is interesting. The “Band suspected” flag on the ProCyte Dx indicates the presence of toxic and/or immature neutrophils (suggesting systemic inflammation) on the basis of the characteristic appearance of the dot plots. Thus, this association suggests there is some relationship between systemic inflammation and RBC resistance to the ProCyte One lysis protocol, although the mechanism by which these two factors might be related is still unclear. Furthermore, the fact that this association is not consistently observed suggests inflammation is likely only one of many factors contributing to RBC lysis resistance. Further investigation as to the cause of the uRBCs and optimization of reagents for more consistent RBC lysis in dogs for this analyzer is needed. Interference from uRBCs was not appreciated in cats, possibly because of differences in the interaction of the lysing reagents with RBCs from different species and/or an easier separation of uRBCs from lymphocytes by light scatter properties because of the smaller RBC size in cats.

The ProCyte One had generally excellent agreement with both analyzers for WBCs and most leukocytes. The most notable exception was monocytes, where a moderate (ProCyte Dx) to large (ADVIA 120) positive proportional bias was present in both cats and dogs. This bias was readily appreciated visually in the Bland–Altman plots (Figures S3, S4, S7, and S8). Although all three analyzers use laser flow cytometry, the method by which leukocyte populations are differentiated varies between them. The ADVIA 120 characterizes cells by size and myeloperoxidase activity, and the ProCyte Dx uses fluorescent stain uptake and internal complexity. In contrast, the ProCyte One uses only light scatter properties to characterize leukocytes. Because of differences in methodology, poor correlation and high bias for monocytes are commonly reported between hematology analyzers in veterinary species [9, 16, 17]. Given the major differences in monocytes between these three analyzers, it is questionable as to which of the three is most “accurate”. When compared to the manual differential, the ProCyte One had a smaller, but still apparent, positive bias for monocyte %, suggesting systematic overestimation of monocytes. However, the manual differential is not a perfect gold standard, as uneven cell distribution throughout the smears can influence results. Furthermore, monocytes can be misidentified even by experienced hematologists because of the high variability in their morphologic features [15]. Regardless of which method is most accurate, the differences in the monocyte results are interesting from an analytical standpoint but are unlikely to be of major clinical significance.

Agreement between analyzers for RBC parameters was generally good, although a few notable differences were seen. The biggest difference was MCV compared to the ADVIA 120, where a large bias caused total error to exceed ASVCP‐recommended TEa in both dogs and cats (Table 5). The mean bias of −6.0 fL in dogs for the ProCyte One (Table 2) indicates that the ADVIA 120, on average, measured 6 fL higher than the ProCyte One. Given the relatively narrow reference interval for MCV (ProCyte One RI 61.6–73.5 fL), a difference of 6 fL could easily affect the classification of the erythron (e.g., microcytic/normocytic/macrocytic) in some patients and alter interpretation. Thus, we recommend that the MCV and values calculated using MCV (e.g., HCT and MCHC) should not be directly compared between the ProCyte One and ADVIA 120. Additionally, although mean bias was relatively low for MCV compared with the ProCyte Dx (0.3 fL and −1.6 fL in dogs and cats, respectively), there were some individual samples with relatively large and potentially clinically important differences, especially in cats (Figure S5). As such, caution should also be applied in comparing MCV, HCT, and MCHC results between the ProCyte One and ProCyte Dx.

Although not the purpose of this study, the magnitude of the MCV bias between the ProCyte One and ADVIA 120 raised interest regarding which method might be more accurate. There is no gold standard method to compare to for MCV, but HCT, which is calculated by multiplying MCV and RBC, can be compared with the manual PCV. Manual PCV versus HCT generally showed close agreement for the ProCyte One (Figure S9a; mean difference 0.1%), but a trend toward positive bias of 2.7% was found for the ADVIA 120 (although the 95% limits of agreement still include 0; Figure S9c). This comparison suggests overestimation of the MCV by the ADVIA 120 used in this study. Although the ADVIA 120 was within QC specifications for MCV for the entire study period, there were several limitations to the QC performed. First, the QCM was run using the manufacturer's recommendation for control limits. Manufacturer's control limits are based on imprecision across many analyzers, which is generally much higher than the imprecision of a single analyzer [18]. This can lead to a poor probability of error detection, meaning medically important errors might be missed. A second limitation is related to the commutability of fixed blood QCM. Although fixed blood QCM is designed to mimic a patient sample, factors such as species sources, fixation effects, age‐related changes, and analyzer methodology can significantly impact the relevance of fixed blood QCM performance to actual patient testing [13]. Thus, results from fixed blood QCM do not always prove adequate performance with patient samples. Unlike the ADVIA 120 in this study, the ProCyte One does not use fixed blood QCM. Instead, it uses a non‐cellular, microbead‐based QCM, which is arguably more disparate from patient samples than a fixed blood QCM. However, the ProCyte One also uses PBA‐QC to evaluate for analytical drift and recalibrate parameters as needed. The close agreement between the ProCyte One HCT and PCV seems to support the validity of this QC method.

Agreement was acceptable for platelets compared with the ADVIA 120, but compared with the ProCyte Dx, total observed error approached (feline) or exceeded (canine) allowable error (Table 5). This finding was surprising because the ProCyte Dx was used as the reference method in designing the ProCyte One. Although the reason for the difference is uncertain, it may be related to their differences in how they enumerate platelets. The ProCyte One uses the properties of light scatter to classify platelets, similar to the ADVIA 120 [11, 16]. In contrast, the ProCyte Dx uses electrical flow impedance in dogs and optical fluorescence in cats [9]. Regardless of the reason, the unacceptably high TEobs indicates that canine platelet results should not be directly compared between the ProCyte One and ProCyte Dx.

One major limitation of this study was the less‐than‐excellent, or in some cases poor, correlation for some measurands, especially MCV, MCHC, lymphocytes, and monocytes. Poor correlation in method comparison studies is generally indicative of a narrow data range, and it's a problem because it limits the reliability of linear regression statistics [19]. One solution would be to include more samples with extreme values in order to widen the data range; however, there are several reasons why this might not help improve correlation for some measurands. First, some analytes, such as MCHC, have an inherently narrow population distribution in both health and disease [10]. Second, results can have a non‐linear relationship because of differences in methodologies, although good correlation could still be achieved in this situation (e.g., by logarithmic transformation of the dataset) as long as the bias affects all samples in a consistent manner [5]. If, however, there is a source of bias affecting only some samples but not others, widening the data range and acquiring more samples might not be sufficient to achieve good correlation. For example, because of its methodology, the ProCyte Dx commonly misclassifies immature neutrophils and neutrophil precursors as lymphocytes and monocytes, resulting in the “Band suspect” flag and a message on the analyzer to confirm with dot plot and/or blood film review [15]. Previous investigators have nonetheless shown excellent correlation between the ProCyte Dx and manual differential, but only by excluding samples with clear evidence of this interference [9]. It is unclear in our study if the observed sub‐optimal correlation for the ProCyte One lymphocyte and monocyte differential is due to an inconsistent source of bias or merely because of an overly narrow data range and insufficient sample numbers. On the basis of evaluation of ProCyte One dot plots, the only repeatable source of error appreciated by the authors was interference from uRBCs; however, excluding samples flagged for uRBCs did not significantly improve the correlation for lymphocytes (data not shown). Regardless of the cause, we elected to use Passing–Bablok regression as previously recommended because it is less affected than simple linear regression by poor correlation [5].

Another limitation was that some comparison data did not display a linear relationship on the basis of the CUSUM statistic, suggesting Passing–Bablok regression was inappropriate (indicated by “a” in Tables 2, 3, 4). This suggests some degree of systematic proportional bias is present [5], although this could not always be appreciated by visual inspection of the data. It was, however, readily apparent in the Bland–Altman plots of RBC compared to both analyzers in dogs, where the trend of the plotted differences appears to curve downwards in more anemic dogs (Figures 2a and 3a). This indicates that the lower the RBC count, the greater the negative bias of the ProCyte One. This finding is concerning, as underestimation of RBC mass in an anemic animal could affect medical decision‐making, especially with regard to blood transfusions. Given the non‐linearity of the data, the ideal solution to better assess proportional bias would be to calculate separate regression lines for smaller subsets of the data [5] (e.g., dogs above or below 5.0 M RBC/μL). Additionally, total error could be reevaluated on each subset to verify that results are comparable regardless of RBC mass. Unfortunately, there were not enough anemic dogs included in this study to perform this analysis. Thus, the regression statistics were reported on the basis of the full data set with the caveat that these analyses are likely not fully representative of the relationship between the analyzers. Further comparison testing with a focus on anemic canine samples is needed.

There were a few additional limitations with our feline cohort. For one, only 5 cats had reticulocytes > 60 000/μL on at least one analyzer. This made meaningful comparison between analyzers impossible since automated reticulocyte counts below 60 000/μL are associated with high imprecision [10]. Additionally, only one feline sample was obtained with enough volume to determine intra‐assay precision. Although most analytes demonstrated excellent precision for this sample, testing of additional samples that span the analytical ranges is warranted.

## 5 Conclusions

This report represents the first external evaluation of the ProCyte One hematology analyzer. In this study, we found that, in dogs and cats, ProCyte One results were directly comparable with results of the ProCyte Dx and the ADVIA 120 for most measurands, with the major exceptions of MCV compared with the ADVIA 120, platelets compared with the ProCyte Dx, and monocytes compared with both. ProCyte One results overall compared favorably to those obtained using manual methods, but manual PCV and blood morphology evaluation are still recommended as part of comprehensive hematology testing because of occasional clinically important differences in some patients. Overall, these findings are promising, but more work is still needed in pursuit of complete independent validation of the ProCyte One according to ASVCP recommendations [4].

## Conflicts of Interest

All instrumentation and reagents used in this study were provided by IDEXX, the manufacturer of the ProCyte One, and additional funding support was provided for laboratory technician time. Additionally, K. Yore and J. Hammond are currently employed, and D. B. Denicola was formerly employed, by IDEXX. M. B. Nabity has received research support and honoraria from IDEXX within the past 5 years.

## Supporting information


**Appendix S1:** vcp70071‐sup‐0001‐AppendixS1.zip.
