# Supplementary material for: Comparison of the IDEXX ProCyte One to the ProCyte Dx and ADVIA 120 in Dogs and Cats
Source: Vet Clin Pathol. 2025 Nov 4;54(4):338–54. doi: 10.1111/vcp.70071 (PMC12885859; doi:10.1111/vcp.70071)
Supplement: Supplementary file 1 — Appendix S1: vcp70071‐sup‐0001‐AppendixS1.zip. [file VCP-54-338-s001.zip › vcp70071-sup-0011-TablesS1-S3.docx]

**Supplemental Table 1:** Manufacturers’ reported linear range and precision of the SIEMENS ADVIA 120, IDEXX ProCyte Dx, and IDEXX ProCyte One

|  |  | ADVIA 120 | | ProCyte Dx | | ProCyte One | |
| --- | --- | --- | --- | --- | --- | --- | --- |
|  |  | Linear Range | CV (%) | Linear Range | CV (%) | Linear Range | CV (%) |
| RBC | M/uL | 0.0-7.0 | 1.2 | 0.0-16.0 | 1.5 | 0-16 | 1.1 |
| Reticulocytes | % | 0.2-24.5 | 12.5 | 0.0-23 | 15^1^ | N/A | 15^1^ |
| Reticulocytes | M/uL | N/A | N/A | 0-3.0 | 15^1^ | 0-3 | 15^1^ |
| Hgb | g/dL | 0.0-22.5 | 0.93 | 0.0-25.0 | 1.5 | 0-25 | 1.3 |
| MCV | fL | N/A | 0.78 | N/A | N/A | N/A | N/A |
| WBC | K/uL | 0.02-400 | 2.7 | 0-310 | 3.0 | 0-310 | 3.4 |
| Platelets | K/uL | 5.0-3500 | 2.93 | 0-2000 | 4.0^2^ | 0-2000 | 3.2 |

RBC – red blood cells; HCT – hematocrit; PCV – packed cell volume; Hgb – hemoglobin; MCV – mean cell volume; WBC – white blood cells; CV – coefficient of variation; N/A – not available

^1^ RBC 3.00 M/uL or more, %Retic 1%-4%

^2^ PLT 100K/uL or more

**Supplemental Table 2:** Descriptive statistics of CBC results in the canine cohort used for method comparison.

|  |  |  |  | **ProCyte One** | | | |  | **ProCyte Dx** | | | |  | **ADVIA 120** | | | |  | | **Manual Methods** | | | | |  |
| --- | --- | --- | --- | --- | --- | --- | --- | --- | --- | --- | --- | --- | --- | --- | --- | --- | --- | --- | --- | --- | --- | --- | --- | --- | --- |
|  | Units | n |  | Mean | Med | Min | Max |  | Mean | Med | Min | Max |  | Mean | Med | Min | Max | |  | | Mean | Med | Min | Max | |
| RBC | M/uL | 140 |  | 6.32 | 6.54 | 1.14 | 9.16 |  | 6.22 | 6.34 | 1.58 | 9.25 |  | 6.18 | 6.23 | 1.67 | 9.73 | |  | |  |  |  |  | |
| HCT/PCV | % | 139 |  | 41.4 | 42.6 | 8.3 | 59.9 |  | 40.6 | 41.0 | 11.6 | 60.1 |  | 44.0 | 44.3 | 13.9 | 66.8 | |  | | 41 | 42 | 12 | 65 | |
| Hgb | g/dL | 130 |  | 14.3 | 14.7 | 3.4 | 20.4 |  | 14.1 | 14.4 | 3.8 | 20.3 |  | 14.3 | 14.6 | 4.1 | 21.3 | |  | |  |  |  |  | |
| MCV | fL | 140 |  | 65.45 | 65.65 | 55.67 | 73.53 |  | 65.19 | 65.15 | 55.50 | 77.20 |  | 71.44 | 71.15 | 64.50 | 89.50 | |  | |  |  |  |  | |
| MCHC | g/dL | 130 |  | 34.94 | 34.79 | 31.89 | 43.16 |  | 35.03 | 35.10 | 31.10 | 37.90 |  | 32.50 | 32.65 | 27.40 | 36.30 | |  | |  |  |  |  | |
| Reticulocytes | k/uL | 140 |  | 51.4 | 30.4 | 0.6 | 403.6 |  | 62.5 | 36.0 | 4.5 | 442.2 |  | 68.1 | 43.0 | 10.1 | 446.4 | |  | |  |  |  |  | |
| WBC | k/uL | 137 |  | 13.13 | 10.97 | 1.61 | 51.22 |  | 13.15 | 10.85 | 1.41 | 67.72 |  | 12.82 | 10.65 | 1.33 | 71.30 | |  | |  |  |  |  | |
| Neutrophils | k/uL | 137 |  | 9.14 | 7.26 | 0.91 | 36.22 |  | 9.27 | 7.60 | 0.19 | 30.10 |  | 9.07 | 7.32 | 0.83 | 38.66 | |  | |  |  |  |  | |
| %Neutrophils | % | 137 |  | 70.2 | 72.1 | 14.2 | 94.1 |  |  |  |  |  |  |  |  |  |  | |  | | 76.0 | 78.5 | 21.0 | 96.5 | |
| Lymphocytes | k/uL | 137 |  | 2.34 | 1.30 | 0.29 | 31.31 |  | 1.89 | 1.39 | 0.26 | 13.75 |  | 2.39 | 1.53 | 0.18 | 45.92 | |  | |  |  |  |  | |
| %Lymphocytes | % | 137 |  | 16.9 | 15.4 | 1.4 | 66.7 |  |  |  |  |  |  |  |  |  |  | |  | | 13.6 | 11.5 | 0.0 | 70.0 | |
| Monocytes | k/uL | 137 |  | 1.28 | 0.84 | 0.06 | 10.58 |  | 1.52 | 0.63 | 0.11 | 41.17 |  | 0.79 | 0.55 | 0.08 | 15.33 | |  | |  |  |  |  | |
| %Monocytes | % | 137 |  | 9.9 | 8.3 | 1.1 | 58.4 |  |  |  |  |  |  |  |  |  |  | |  | | 6.3 | 5.0 | 0.0 | 37.0 | |
| Eosinophils | k/uL | 137 |  | 0.28 | 0.20 | 0.00 | 1.80 |  | 0.35 | 0.26 | 0.00 | 2.17 |  | 0.31 | 0.21 | 0.01 | 2.04 | |  | |  |  |  |  | |
| %Eosinophils | % | 137 |  | 3.0 | 2.2 | 0.0 | 26.8 |  |  |  |  |  |  |  |  |  |  | |  | | 3.6 | 2.5 | 0.0 | 27.0 | |
| Platelets | k/uL | 117 |  | 289 | 270 | 12 | 1039 |  | 327 | 294 | 0 | 1156 |  | 305 | 262 | 4 | 1018 | |  | |  |  |  |  | |

Med – median; Min – minimum; Max – maximum; RBC – red blood cells; HCT – hematocrit; PCV – packed cell volume; Hgb – hemoglobin; MCV – mean corpuscular volume; MCHC – mean corpuscular hemoglobin concentration; WBC – white blood cells

**Supplemental Table 3**: Descriptive statistics of CBC results in feline cohort used for method comparison

|  |  |  |  | **ProCyte One** | | | |  | **ProCyte Dx** | | | |  | **ADVIA 120** | | | |  | | **Manual Methods** | | | | |
| --- | --- | --- | --- | --- | --- | --- | --- | --- | --- | --- | --- | --- | --- | --- | --- | --- | --- | --- | --- | --- | --- | --- | --- | --- |
|  | Units | n |  | Mean | Med | Min | Max |  | Mean | Med | Min | Max |  | Mean | Med | Min | Max |  | Mean | | Med | Min | Max |  |
| RBC | M/uL | 95 |  | 8.14 | 8.16 | 2.82 | 12.92 |  | 7.91 | 7.81 | 2.64 | 14.11 |  | 7.76 | 7.84 | 2.82 | 13.58 |  |  | |  |  |  |  |
| HCT/PCV | % | 93 |  | 35.1 | 36.4 | 11.5 | 54.6 |  | 35.3 | 35.8 | 11.5 | 62.8 |  | 35.6 | 36.6 | 12.9 | 65.1 |  | 34 | | 35 | 10 | 62 |  |
| Hgb | g/dL | 92 |  | 11.6 | 11.9 | 3.3 | 19.7 |  | 11.5 | 11.6 | 3.7 | 20.0 |  | 11.6 | 11.9 | 5.1 | 19.9 |  |  | |  |  |  |  |
| MCV | fL | 95 |  | 43.60 | 43.51 | 29.55 | 53.77 |  | 45.19 | 44.50 | 34.70 | 57.60 |  | 46.39 | 46.30 | 36.70 | 54.70 |  |  | |  |  |  |  |
| MCHC | g/dL | 92 |  | 33.00 | 32.95 | 27.37 | 42.53 |  | 32.37 | 32.45 | 27.50 | 37.40 |  | 32.19 | 32.25 | 30.10 | 34.10 |  |  | |  |  |  |  |
| Reticulocytes | k/uL | 95 |  | 15.2 | 9.3 | 0.2 | 169.9 |  | 19.9 | 12.3 | 2.5 | 191.3 |  | 20.9 | 15.9 | 3.5 | 150.7 |  |  | |  |  |  |  |
| WBC | k/uL | 95 |  | 11.13 | 8.67 | 1.03 | 38.27 |  | 11.77 | 9.65 | 1.37 | 33.75 |  | 11.59 | 9.37 | 1.07 | 34.34 |  |  | |  |  |  |  |
| Neutrophils | k/uL | 95 |  | 8.00 | 5.41 | 0.61 | 32.05 |  | 7.74 | 6.02 | 0.18 | 24.62 |  | 88.00 | 6.15 | 0.85 | 30.18 |  |  | |  |  |  |  |
| %Neutrophils | % | 88 |  | 66.3 | 67.3 | 26.3 | 92.9 |  |  |  |  |  |  |  |  |  |  |  | 71.7 | | 72.3 | 34.5 | 99.5 |  |
| Lymphocytes | k/uL | 95 |  | 2.11 | 1.90 | 0.31 | 9.45 |  | 3.04 | 2.21 | 0.09 | 24.74 |  | 2.59 | 2.14 | 0.16 | 12.51 |  |  | |  |  |  |  |
| %Lymphocytes | % | 88 |  | 23.1 | 20.0 | 2.9 | 66.8 |  |  |  |  |  |  |  |  |  |  |  | 20.2 | | 16.8 | 0.0 | 63.5 |  |
| Monocytes | k/uL | 95 |  | 0.62 | 0.50 | 0.05 | 2.35 |  | 0.51 | 0.37 | 0.05 | 4.80 |  | 0.28 | 0.23 | 0.02 | 1.21 |  |  | |  |  |  |  |
| %Monocytes | % | 88 |  | 5.9 | 5.4 | 0.9 | 18.2 |  |  |  |  |  |  |  |  |  |  |  | 3.4 | | 3.0 | 0.0 | 11.0 |  |
| Eosinophils | k/uL | 95 |  | 0.37 | 0.27 | 0.03 | 1.38 |  | 0.38 | 0.28 | 0.00 | 1.30 |  | 0.33 | 0.28 | 0.00 | 1.18 |  |  | |  |  |  |  |
| %Eosinophils | % | 88 |  | 4.3 | 3.6 | 0.2 | 17.3 |  |  |  |  |  |  |  |  |  |  |  | 4.3 | | 3.0 | 0.0 | 17.0 |  |
| Platelets | k/uL | 56 |  | 247 | 250 | 23 | 612 |  | 273 | 271 | 20 | 658 |  | 246 | 246 | 29 | 587 |  |  | |  |  |  |  |

Med – median; Min – minimum; Max – maximum; RBC – red blood cells; HCT – hematocrit; PCV – packed cell volume; Hgb – hemoglobin; MCV – mean corpuscular volume; MCHC – mean corpuscular hemoglobin concentration; WBC – white blood cells
