# Supplementary material for: Comparison of the IDEXX ProCyte One to the ProCyte Dx and ADVIA 120 in Dogs and Cats
Source: Vet Clin Pathol. 2025 Nov 4;54(4):338–54. doi: 10.1111/vcp.70071 (PMC12885859; doi:10.1111/vcp.70071)
Supplement: Supplementary file 1 — Appendix S1: vcp70071‐sup‐0001‐AppendixS1.zip. [file VCP-54-338-s001.zip › misc/VCP-24-4459.R2 Supplementary Figure Legends.docx]

**Fig S1:** Canine automated CBC comparison between ProCyte One (PC One) and ProCyte Dx (PC Dx). *Left graphs:* scatter plots with Passing-Bablok regression line (solid blue line) and confidence interval (dashed blue lines). Black is line of identity. *Right graphs:* Bland-Altman plots with mean (solid blue line) +/- 1.96 SD (dashed blue lines). **a.** Comparison of HCT; **b.** Comparison of Hgb; **c.** Comparison of MCV; **d.** Comparison of MCHC.

**Fig S2:** Canine automated CBC comparison between ProCyte One (PC One) and ADVIA 120 (ADVIA). *Left graphs:* scatter plots with Passing-Bablok regression line (solid blue line) and confidence interval (dashed blue lines). Black is line of identity. *Right graphs:* Bland-Altman plots with mean (solid blue line) +/- 1.96 SD (dashed blue lines). **a.** Comparison of HCT; **b.** Comparison of Hgb; **c.** Comparison of MCV; **d.** Comparison of MCHC.

**Fig S3:** Canine automated leukocyte count comparison between ProCyte One (PC One) and ProCyte Dx (PC Dx). *Left graphs:* scatter plots with Passing-Bablok regression line (solid blue line) and confidence interval (dashed blue lines). Black is line of identity. *Right graphs:* Bland-Altman plots with mean (solid blue line) +/- 1.96 SD (dashed blue lines). Red data points are runs where the result was flagged by the ProCyte One, the ProCyte Dx, or both. **a.** Comparison of automated neutrophils; **b.** Comparison of automated lymphocytes; **c.** Comparison of automated monocytes; **d.** Comparison of automated eosinophils.

**Fig S4:** Canine automated leukocyte count comparison between ProCyte One (PC One) and ADVIA 120 (ADVIA). *Left graphs:* scatter plots with Passing-Bablok regression line (solid blue line) and confidence interval (dashed blue lines). Black is line of identity. *Right graphs:* Bland-Altman plots with mean (solid blue line) +/- 1.96 SD (dashed blue lines). Red data points are runs where the result was flagged by the ProCyte One, the ProCyte Dx, or both. **a.** Comparison of automated neutrophils; **b.** Comparison of automated lymphocytes; **c.** Comparison of automated monocytes; **d.** Comparison of automated eosinophils.

**Fig S5:** Feline automated CBC comparison between ProCyte One (PC One) and ProCyte Dx (PC Dx). *Left graphs:* scatter plots with Passing-Bablok regression line (solid blue line) and confidence interval (dashed blue lines). Black is line of identity. *Right graphs:* Bland-Altman plots with mean (solid blue line) +/- 1.96 SD (dashed blue lines). **a.** Comparison of HCT; **b.** Comparison of Hgb; **c.** Comparison of MCV; **d.** Comparison of MCHC.

**Fig S6:** Feline automated CBC comparison between ProCyte One (PC One) and ADVIA 120 (ADVIA). *Left graphs:* scatter plots with Passing-Bablok regression line (solid blue line) and confidence interval (dashed blue lines). Black is line of identity. *Right graphs:* Bland-Altman plots with mean (solid blue line) +/- 1.96 SD (dashed blue lines). **a.** Comparison of HCT; **b.** Comparison of Hgb; **c.** Comparison of MCV; **d.** Comparison of MCHC.

**Fig S7:** Feline automated leukocyte count comparison between ProCyte One (PC One) and ProCyte Dx (PC Dx). *Left graphs:* scatter plots with Passing-Bablok regression line (solid blue line) and confidence interval (dashed blue lines). Black is line of identity. *Right graphs:* Bland-Altman plots with mean (solid blue line) +/- 1.96 SD (dashed blue lines). Red data points are runs where the result was flagged by the ProCyte One, the ProCyte Dx, or both. **a.** Comparison of automated neutrophils; **b.** Comparison of automated lymphocytes; **c.** Comparison of automated monocytes; **d.** Comparison of automated eosinophils.

**Fig S8:** Feline automated leukocyte count comparison between ProCyte One (PC One) and ADVIA 120 (ADVIA). *Left graphs:* scatter plots with Passing-Bablok regression line (solid blue line) and confidence interval (dashed blue lines). Black is line of identity. *Right graphs:* Bland-Altman plots with mean (solid blue line) +/- 1.96 SD (dashed blue lines). Red data points are runs where the result was flagged by the ProCyte One, the ProCyte Dx, or both. **a.** Comparison of automated neutrophils; **b.** Comparison of automated lymphocytes; **c.** Comparison of automated monocytes; **d.** Comparison of automated eosinophils.

**Fig S9:** Canine comparison of HCT for each analyzer to manual PCV. *Left graphs:* scatter plots with Passing-Bablok regression line (solid blue line) and confidence interval (dashed blue lines). Black is line of identity. *Right graphs:* Bland-Altman plots with mean (solid blue line) +/- 1.96 SD (dashed blue lines). **a.** Comparison of ProCyte One; **b.** Comparison of ProCyte Dx; **c.** Comparison of ADVIA 120.

**Fig S10:** Feline comparison of HCT for each analyzer to manual PCV. *Left graphs:* scatter plots with Passing-Bablok regression line (solid blue line) and confidence interval (dashed blue lines). Black is line of identity. *Right graphs:* Bland-Altman plots with mean (solid blue line) +/- 1.96 SD (dashed blue lines). **a.** Comparison of ProCyte One; **b.** Comparison of ProCyte Dx; **c.** Comparison of ADVIA 120.
