# Supplementary material for: Comparison of the IDEXX ProCyte One to the ProCyte Dx and ADVIA 120 in Dogs and Cats
Source: Vet Clin Pathol. 2025 Nov 4;54(4):338–54. doi: 10.1111/vcp.70071 (PMC12885859; doi:10.1111/vcp.70071)
Supplement: Supplementary file 1 — Appendix S1: vcp70071‐sup‐0001‐AppendixS1.zip. [file VCP-54-338-s001.zip › vcp70071-sup-0012-TableS4.docx]

**Supplemental Table 4:** Comparison data with and without flagged data points in dogs

| Measurand |  | n |  | ProCyte Dx | | | |  | ADVIA 120 | | | |
| --- | --- | --- | --- | --- | --- | --- | --- | --- | --- | --- | --- | --- |
|  | Unit |  |  | ρ | Slope  (95% CI) | Intercept  (95% CI) | Bias  (95% LOA) |  | ρ | Slope  (95% CI) | Intercept  (95% CI) | Bias  (95% LOA) |
| WBC | 10^9^/L | 137 |  | 0.99 | 1.00  (0.98-1.02) | -0.1  (-0.3, 0.1) | 0.0  (-5.9, 5.8) |  | 0.99 | 1.03  (1.00-1.05) | 0.0  (-0.3, 0.2) | 0.3  (-6.1, 6.8) |
| WBC excluding flags | 10^9^/L | 103 |  | 0.99 | 0.99  (0.96-1.01) | -0.1  (-0.3, 0.2) | -0.1  (-1.3, 1.0) |  | 0.99 | 1.02  (0.99-1.04) | 0.0  (-0.2, 0.3) | 0.2  (-1.0, 1.4) |
| Neutrophils | 10^9^/L | 137 |  | 0.98 | 1.00  (0.99-1.02) | **-0.2**  **(-0.4, -0.1)** | -0.1  (-2.4, 2.1) |  | 0.96 | 1.02  (1.00-1.03) | 0.0  (-0.1, 0.1) | 0.1  (-2.1, 2.2) |
| Neutrophils excluding flags | 10^9^/L | 115 |  | 1.00 | 0.99  (0.98-1.01) | -0.2  (-0.2, 0.0) | -0.2  (-0.9, 0.7) |  | 0.99 | 1.02  (1.00-1.04) | 0.0  (-0.1, 0.1) | 0.1  (-1.5, 0.9) |
| Lymph | 10^9^/L | 137 |  | 0.78 | 1.09  (0.98-1.24) | -0.1  (-0.4, 0.0) | 0.4  (-5.4, 6.3) |  | 0.72 | 1.04  (0.93-1.09) | -0.2  (-0.4, 0.0) | -0.1  (-5.6, 5.5) |
| Lymph excluding flags | 10^9^/L | 97 |  | 0.79 | 1.00  (0.92-1.01) | -0.1  (-0.2, 0.1) | -0.1  (-1.0, 0.7) |  | 0.81 | 0.98  (0.90-1.09) | -0.1  (-0.3, 0.0) | -0.2  (-0.8, 0.6) |
| Monocytes | 10^9^/L | 137 |  | 0.91 | **1.32**  **(1.23-1.47)** | 0.0  (-0.1, 0.1) | -0.2  (-7.8, 7.3) |  | 0.91 | **2.05**  **(1.91-2.21)** | **-0.2**  **(-0.3, -0.1)** | 0.5  (-2.1, 3.1) |
| Monocytes excluding flags | 10^9^/L | 114 |  | 0.93 | **1.47**  **(1.33-1.69)** | 0.0  (-0.1, 0.0) | 0.3  (-1.6, 1.0) |  | 0.91 | **2.02**  **(1.88-2.17)** | **-0.2**  **(-0.2, -0.1)** | 0.5  (-2.5, 1.5) |

WBC – white blood cells; n – number of samples; ρ – Spearman’s rho; CI – confidence interval; LoA – limits of agreement

Bolded results for Slope indicate the 95% CI does not include 1, suggesting proportional bias is present

Bolded results for Intercept indicate the 95% CI does not include 0, suggesting constant bias is present
