# Supplementary figures and images for: Comparison of the IDEXX ProCyte One to the ProCyte Dx and ADVIA 120 in Dogs and Cats
Source: Vet Clin Pathol. 2025 Nov 4;54(4):338–54. doi: 10.1111/vcp.70071 (PMC12885859; doi:10.1111/vcp.70071)

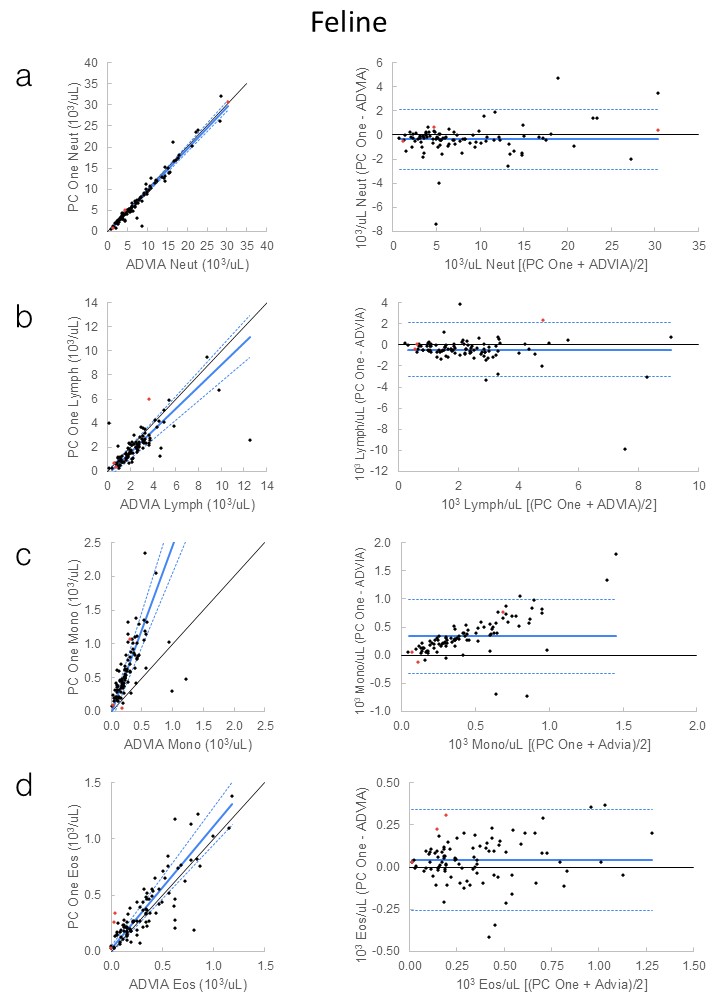

Supplement: Supplementary file 1 — Appendix S1: vcp70071‐sup‐0001‐AppendixS1.zip. [file VCP-54-338-s001.zip › vcp70071-sup-0008-FigureS8.jpg]

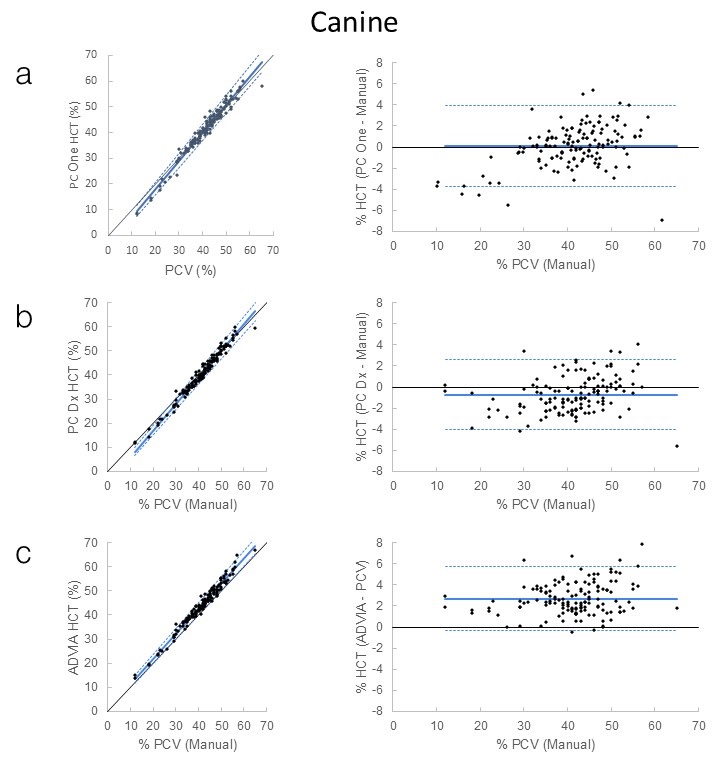

Supplement: Supplementary file 1 — Appendix S1: vcp70071‐sup‐0001‐AppendixS1.zip. [file VCP-54-338-s001.zip › vcp70071-sup-0009-FigureS9.jpg]

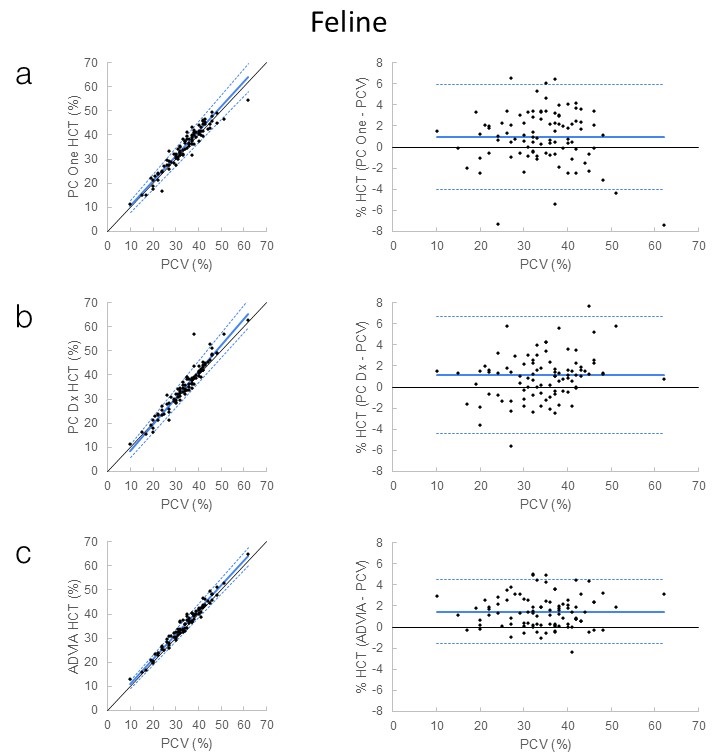

Supplement: Supplementary file 1 — Appendix S1: vcp70071‐sup‐0001‐AppendixS1.zip. [file VCP-54-338-s001.zip › vcp70071-sup-0010-FigureS10.jpg]

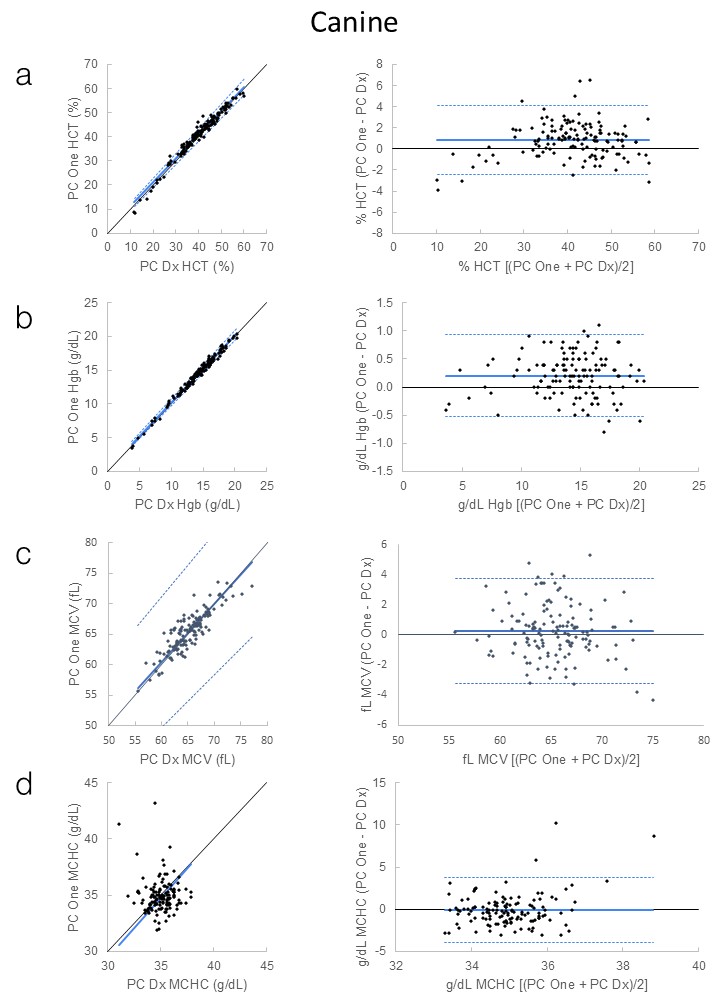

Supplement: Supplementary file 1 — Appendix S1: vcp70071‐sup‐0001‐AppendixS1.zip. [file VCP-54-338-s001.zip › vcp70071-sup-0001-FigureS1.jpg]

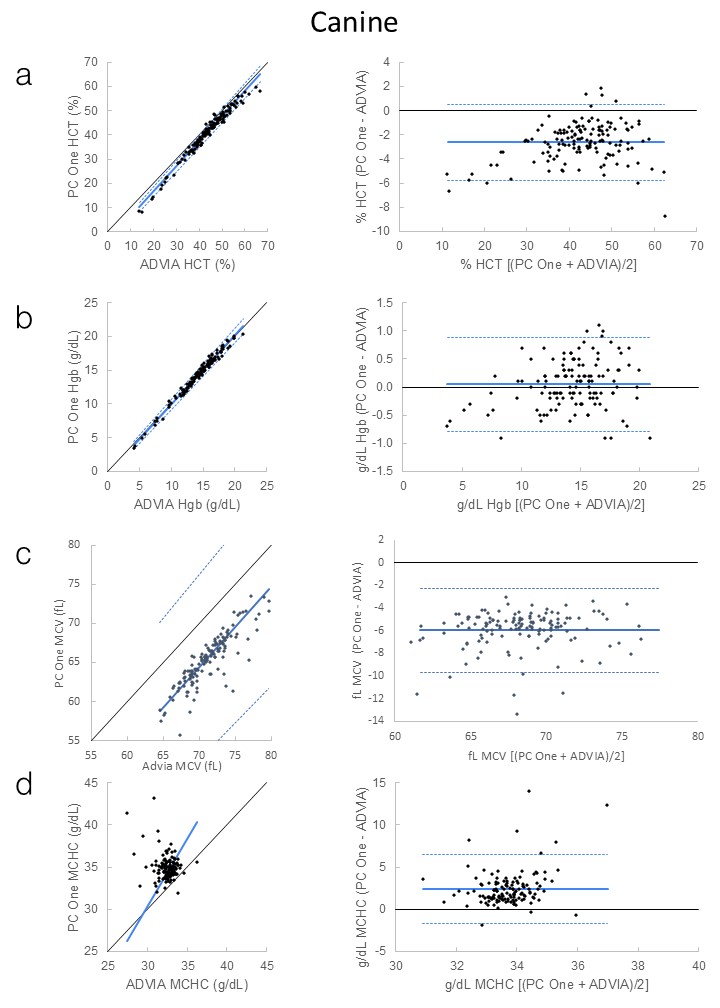

Supplement: Supplementary file 1 — Appendix S1: vcp70071‐sup‐0001‐AppendixS1.zip. [file VCP-54-338-s001.zip › vcp70071-sup-0002-FigureS2.jpg]

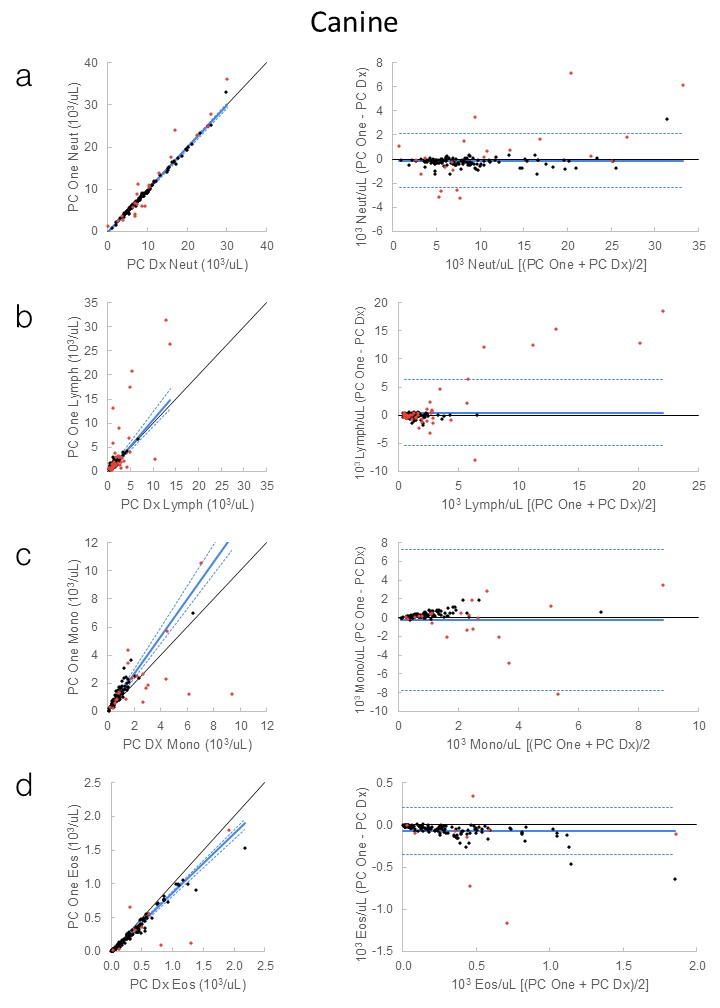

Supplement: Supplementary file 1 — Appendix S1: vcp70071‐sup‐0001‐AppendixS1.zip. [file VCP-54-338-s001.zip › vcp70071-sup-0003-FigureS3.jpg]

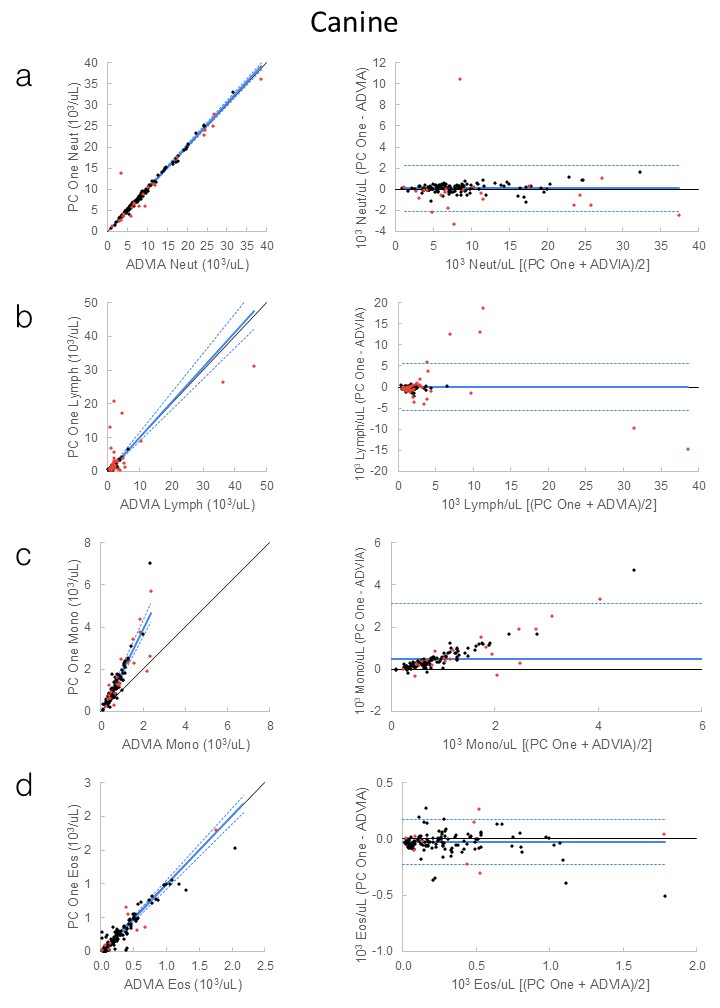

Supplement: Supplementary file 1 — Appendix S1: vcp70071‐sup‐0001‐AppendixS1.zip. [file VCP-54-338-s001.zip › vcp70071-sup-0004-FigureS4.jpg]

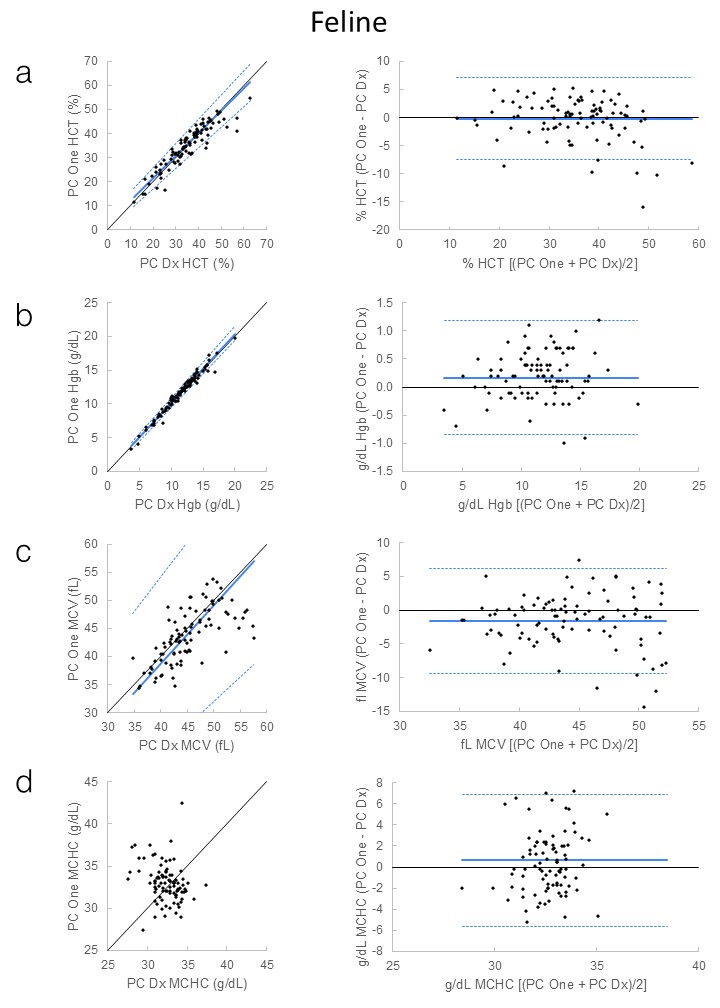

Supplement: Supplementary file 1 — Appendix S1: vcp70071‐sup‐0001‐AppendixS1.zip. [file VCP-54-338-s001.zip › vcp70071-sup-0005-Figure 5.jpg]

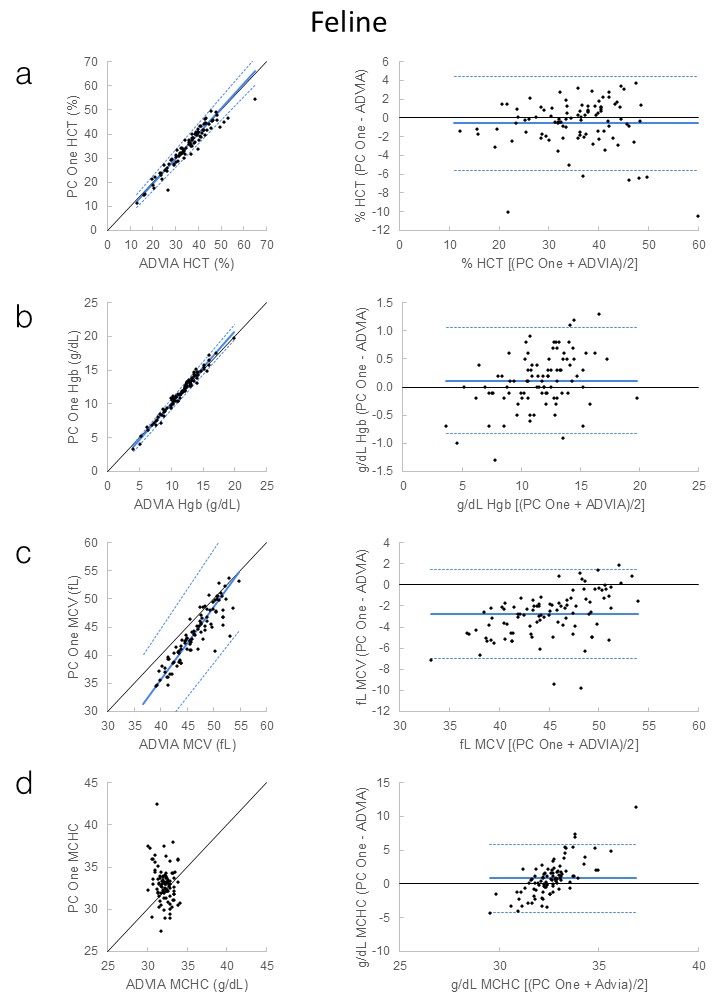

Supplement: Supplementary file 1 — Appendix S1: vcp70071‐sup‐0001‐AppendixS1.zip. [file VCP-54-338-s001.zip › vcp70071-sup-0006-FigureS6.jpg]

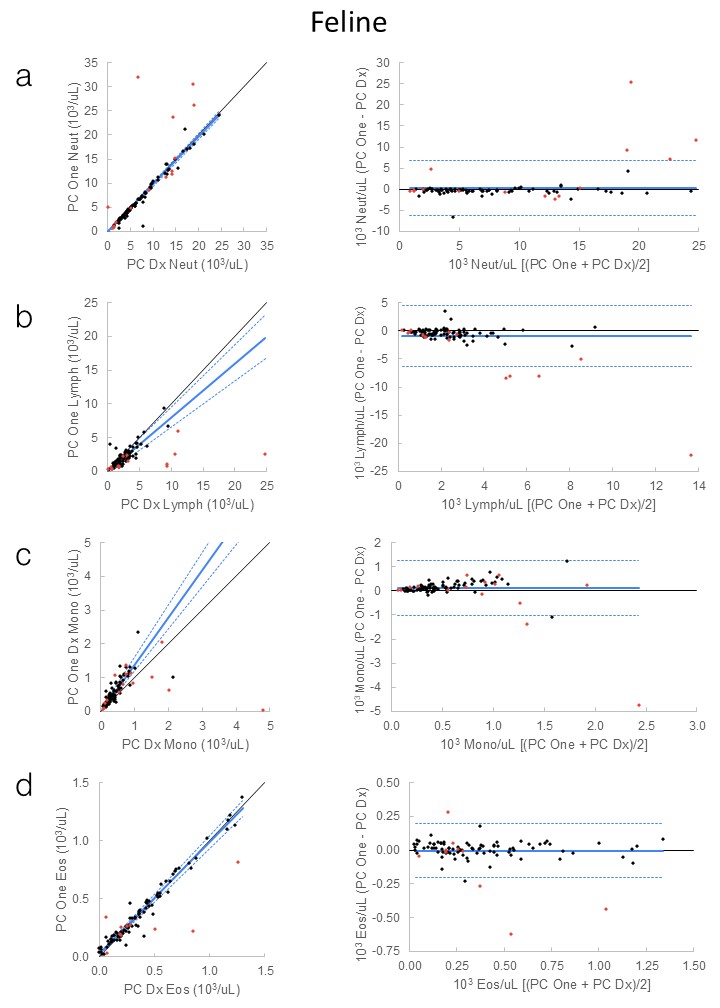

Supplement: Supplementary file 1 — Appendix S1: vcp70071‐sup‐0001‐AppendixS1.zip. [file VCP-54-338-s001.zip › vcp70071-sup-0007-FigureS7.jpg]
